# Long-Term Add-On Use of a Fixed Multicomponent Formulation Containing Palmitoylethanolamide and Acetyl-L-Carnitine in Fibromyalgia Syndrome: A 15-Month Retrospective Single-Cohort Study of 150 Patients

**DOI:** 10.3390/nu18213693

**Published:** 2026-11-07

**Authors:** Enrico Polati, Marco Anderloni, Leonardo Gottin, Katia Donadello, Lorenzo Peluso, Alvise Martini, Luca Polati, Vincenzo Di Maria, Libera Villagrossi, Alex Patton, Filomena Puntillo, Gabriele Finco, Giovanna Del Balzo, Vittorio Schweiger

**Affiliations:** 1 Department of Surgery, Dentistry, Gynecology and Pediatrics, Section of Anaesthesia, Intensive Care and Pain Therapy, University of Verona, 37129 Verona, Italy; enrico.polati@univr.it (E.P.); vittorio.schweiger@univr.it (V.S.); 2 Department of Interdisciplinary Medicine–Pain and ICU Section, University of Bari Aldo Moro, 70121 Bari, Italy; 3 Department of Medical Sciences and Public Health, University of Cagliari, 09124 Cagliari, Italy; 4 Department of Diagnostics and Public Health, Section of Forensic Medicine, University of Verona, 37129 Verona, Italy

**Keywords:** fibromyalgia, palmitoylethanolamide, acetyl-L-carnitine, nutraceuticals, nociplastic pain, sleep quality, real-world evidence, long-term treatment

## Abstract

**Background/Objectives:** Long-term treatment options for fibromyalgia syndrome (FMS) remain limited and there is a lack of real-world data on the extended use of nutraceutical formulations containing palmitoylethanolamide (PEA) and acetyl-L-carnitine (ALC). This study describes a cohort treated for 12 months with a fixed multicomponent formulation containing PEA and ALC, followed by an additional visit three months post-treatment. **Methods:** This retrospective, uncontrolled single-cohort study involved 150 consecutive adults diagnosed with fibromyalgia syndrome (FMS). Participants received a fixed oral formulation comprising palmitoylethanolamide (PEA) 600 mg, acetyl-L-carnitine (ALC) 500 mg, Boswellia serrata 100 mg, vitamin B6 and vitamin E, administered twice daily as an adjunct to stable background therapy. Pain levels were assessed using the Visual Analogue Scale (VAS), and the impact of the disease was evaluated with the revised Fibromyalgia Impact Questionnaire (FIQR) over a period of up to 15 months. Sleep quality was measured using the Pittsburgh Sleep Quality Index (PSQI) for up to 6 months. Missing data were addressed through various methods, including a mixed model, last observation carried forward, delta-adjusted and baseline-carried-forward scenarios, as well as non-responder imputation. **Results:** Sixty-four patients completed the 12-month course, 45 discontinued (36 for perceived lack of efficacy) and 41 were lost to follow-up. Estimated mean VAS fell from 76.3 to 52.3 at 12 months and FIQR from 73.6 to 56.1; under the worst-case assumption that every non-completer returned to baseline, the mean 12-month change in VAS was −13.0 points (95% CI −15.9 to −10.2). At 6 months, 78.1% of completers reached the FIQR minimal clinically important difference and 59.4% the IMMPACT 30% pain threshold, corresponding to 37.3% and 28.7% of all 150 patients with non-responder imputation; only 20.3% of completers (10.0% of all patients) improved by ≥3 PSQI points. Recorded adverse events occurred in 10.0% of patients. Retention at 12 months was 60.6% (42.7% if patients lost to follow-up were counted as discontinuations). In completers, pain and FIQR three months after the end of treatment were within one point of the 12-month values. **Conclusions:** In this uncontrolled cohort, 12 months of add-on treatment with a multicomponent formulation containing PEA and ALC was associated with reductions in pain and disease impact, whereas sleep outcomes changed minimally. However, attrition was substantial and partially response-dependent; therefore, the observed changes cannot be attributed to the formulation or its individual components. These descriptive findings support the need for a controlled trial with a duration of at least 12 months.

## 1. Introduction

Fibromyalgia syndrome (FMS) is a chronic condition characterised by widespread musculoskeletal pain associated with non-restorative sleep, fatigue and cognitive symptoms, and is currently diagnosed according to the 2016 revision of the American College of Rheumatology (ACR) criteria [1]; in the absence of validated biomarkers, the diagnosis remains clinical [2]. The prevalence of FMS symptoms in the general population is estimated at 2–4% [3], with a marked female predominance and a substantial burden of fatigue, sleep disturbance and cognitive complaints [4,5]. FMS is now regarded as the prototypical nociplastic pain disorder, in which augmented central processing of nociceptive and sensory input predominates over identifiable tissue or nerve damage [6,7,8], and its aetiopathogenesis is increasingly conceptualised as an interaction between central sensitisation, peripheral small-fibre alterations, neuroinflammatory mechanisms and psychosocial factors [9].

Management involves multiple approaches. In the updated recommendations, an individualised, integrated care pathway is advocated, in which non-pharmacological measures are first-line and pharmacological options play an adjunctive role [2,10]. Differences between international guidelines on drug management have been analysed in formal comparisons [11]. Even for aerobic exercise, the most supported intervention, the absolute improvement in pain intensity is about 11% and is based on low-quality evidence [12]. The evidence for drugs is even less promising. A review of 21 Cochrane reviews covering 87 trials and 17,631 patients found only duloxetine, milnacipran, and pregabalin have good evidence for pain relief, benefiting about one in ten patients, with no evidence beyond six months [13]. For serotonin–noradrenaline reuptake inhibitors (SNRIs), the number needed to treat for 50% pain relief is 11, with no effect on sleep and a twofold increase in withdrawals due to side effects versus placebo [14]. Pregabalin benefits 10% more patients than placebo but causes dizziness, sleepiness, and weight gain [15]. Since FMS is lifelong, sustainable interventions are valuable.

The growing interest in dietary and nutraceutical strategies arises directly from this therapeutic gap. Dietary composition appears to influence symptom burden: in a randomised study, a personalised Mediterranean diet improved disability, fatigue and anxiety scores in patients with FMS compared with a general balanced diet [16]. Nutraceuticals with a plausible biological rationale, favourable tolerability and costs compatible with prolonged use are therefore attractive candidates for the long-term adjunctive role that conventional pharmacological agents fulfil only partially.

Among the nutraceuticals proposed for FMS, two compounds with complementary mechanisms of action, palmitoylethanolamide (PEA) and acetyl-L-carnitine (ALC), have attracted the greatest clinical interest and have both been studied at our centre [17,18].

PEA is a natural fatty acid amide that reduces mast-cell activation and glial reactivity, mainly through peroxisome proliferator-activated receptor-α and by enhancing endocannabinoid signalling [19,20]. Its long-term effectiveness in fibromyalgic patients was demonstrated in a retrospective series of 407 patients treated as an add-on for 15 months [17].

ALC facilitates the transport of long-chain fatty acids into mitochondria and additionally acts as an epigenetic modulator of nociception: repeated administration induces analgesia by up-regulating type-2 metabotropic glutamate receptors in the spinal cord and dorsal root ganglia through acetylation of the NF-κB p65 subunit [21]. ALC also displays antidepressant properties, as supported by a meta-analysis of 12 randomised trials in which it reduced depressive symptoms with an effect comparable to that of conventional antidepressants and fewer adverse effects [22,23].

The rationale for combining PEA and ALC is based on the complementarity of their mechanisms of action, with PEA acting predominantly on mast cells and glia and ALC on mitochondrial energy metabolism, neuronal trophism and glutamatergic transmission. A fixed combination of PEA and ALC has been shown to improve pain and function in peripheral neuropathies secondary to rheumatic diseases over six months [24] and, in another formulation that also contained Boswellia serrata, vitamin E and vitamin B6, to improve traumatic acute low back pain over eight weeks [25]. In FMS, the only randomised study of the combination enrolled 142 patients stabilised on duloxetine and pregabalin and randomised them either to continue that regimen or to add PEA 600 mg plus ALC 500 mg twice daily for 12 weeks; the combination group showed significantly greater improvement in the Widespread Pain Index, the revised Fibromyalgia Impact Questionnaire (FIQR) and the modified Fibromyalgia Assessment Status [26].

Beyond this 12-week trial, the only longer-term description of the combination in FMS is a retrospective analysis of 77 patients from the Italian Fibromyalgia Registry, assessed with disease-severity questionnaires at 12 and 24 months and restricted to patients who attended both follow-up visits [27]; pain intensity, sleep, health-related quality of life, retention on treatment and the course after discontinuation have not been described over a prolonged period. We therefore describe the trajectory of pain, disease impact, sleep quality and health-related quality of life over 12 months of treatment, and at a further visit three months after its discontinuation, in a consecutive cohort of patients with FMS who received a fixed multicomponent formulation containing PEA and ALC (together with Boswellia serrata, vitamin B6 and vitamin E) as an adjunct to existing therapy in a tertiary pain centre, together with the associated safety profile and treatment retention.

## 2. Materials and Methods

### 2.1. Study Design and Setting

This was a retrospective, observational, single-cohort study of consecutive patients with FMS who received a fixed multicomponent formulation containing PEA and ALC as an add-on to their ongoing standard care. Patients were treated at the Pain Therapy Unit of the Azienda Ospedaliera Universitaria Integrata di Verona. Treatment was started between May 2024 and May 2025; follow-up visits, scheduled up to 15 months after the start of treatment, continued until August 2026, when the database was closed and the records were reviewed for this analysis. No control group was included; the study describes the longitudinal course of symptoms within a single treated cohort and does not compare treatment strategies. The study is reported in accordance with the Strengthening the Reporting of Observational Studies in Epidemiology (STROBE) statement for cohort studies [28].

### 2.2. Participants

A total of 150 consecutive patients were studied. Eligible patients were adults (≥18 years) with a diagnosis of FMS established according to the 2016 revision of the ACR criteria [1], with pain lasting more than three months, who had been prescribed the formulation as an add-on to their existing therapy, and for whom a baseline assessment was available.

Duration of the disease, the presence of other painful conditions (such as osteoarthritis or inflammatory rheumatic diseases), participation in structured exercise or physiotherapy programmes, and the use of psychological interventions, including cognitive behavioural therapy, are not recorded in the Registry and are therefore not available for this cohort. All patients were referred to a tertiary pain centre after the failure of first-line management, which accounts for the high baseline pain intensity and the frequent use of opioids described below.

Baseline concomitant treatment for FMS consisted of stable standard-of-care pharmacotherapy, including one or more of the following drugs: 74% tricyclic antidepressants, 62% gabapentinoids, 46% non-steroidal anti-inflammatory drugs (NSAIDs), 43% acetaminophen, 41% opioids, and 38% muscle relaxants. This was maintained across all enrolled patients at constant dosages for at least four weeks prior to the index visit. Changes in concomitant therapy during follow-up were not systematically recorded; however, patients were instructed to maintain their regimens stable throughout the observation period. Escalation of concomitant therapy because of clinical worsening was recorded as treatment failure and counted as a discontinuation of the study formulation. This pragmatic definition is partly circular with respect to symptom worsening and makes changes in background therapy an unmeasured, time-varying confounder; both points are discussed in Section 4.6.

### 2.3. Intervention

All patients received a fixed oral multicomponent formulation containing, per sachet of granules for oral suspension, PEA 600 mg, ALC 500 mg, Boswellia serrata extract 100 mg, vitamin B6 1.4 mg and vitamin E 12 mg, taken as one sachet twice daily. The formulation was prescribed for 12 months, as an adjunct to, and not as a replacement for, ongoing pharmacological and non-pharmacological management of FMS. The final assessment at 15 months was scheduled three months after the planned end of treatment, in order to evaluate whether the changes observed during treatment persisted after its discontinuation. Adherence was assessed at each visit by patient report; no pill count or dosing diary was available.

The formulation is a commercially available food supplement (Kalanit^®^, Chiesi Italia S.p.A., Parma, Italy), prescribed in routine practice and purchased by the patients. The manufacturer had no role in the design, conduct, analysis, or reporting of the study and did not supply the product. Throughout this article, the product is referred to as “the formulation”; the outcomes observed cannot be attributed to PEA and ALC specifically, since Boswellia serrata, vitamin B6, and vitamin E were co-administered to every patient.

### 2.4. Variables and Data Sources

Demographic and clinical variables were extracted from clinical records. The primary outcome variable was pain intensity, measured as the mean daily score on the visual analogue scale (VAS; 0 = no pain, 100 = worst imaginable pain). Secondary outcome variables were disease impact, measured using the Italian version of the FIQR [29,30] (0–100, higher scores indicating greater impact); sleep quality, measured with the Pittsburgh Sleep Quality Index (PSQI; 0–21, higher scores indicating poorer sleep, a global score ≥ 5 being consistent with poor sleep quality) [31]; and health-related quality of life, measured with the Physical and Mental Component Summary scores of the 12-item Short-Form Health Survey (SF-12 PCS and MCS, higher scores indicating better health) [32].

VAS and FIQR were administered at baseline, at 1, 2, 3, 6, 9, and 12 months during treatment, and at 15 months (three months after discontinuation), following the institutional schedule for chronic pain patients, as used in two previous series from this centre [17,18]. PSQI, SF-12 PCS, and SF-12 MCS were administered at baseline and at 3 and 6 months only. Patients additionally rated their overall satisfaction with treatment on a 7-point Likert scale and reported which symptom domains, if any, they perceived as improved.

Safety variables comprised all adverse events spontaneously reported by patients or recorded by the treating physician, together with treatment discontinuation, the month in which it occurred, and its stated reason (lack of perceived efficacy, adverse event, protocol violation, or other). In the source dataset, an empty entry in the adverse-event field denoted that no adverse event had occurred.

### 2.5. Analysis Populations and Handling of Missing Data

Three analysis populations are reported together. This allows the results to be seen both for patients assessed at each visit and for the entire group.

Observed-case population (primary analysis). All assessments recorded at each time point were analysed without imputation. Values present in the source dataset after a documented discontinuation (carried forward from the last visit) were not treated as observations. The number of patients contributing an observed pain assessment was 150, 128, 111, 92, 80, 71, 64, and 64 at baseline and at 1, 2, 3, 6, 9, 12, and 15 months, respectively.

Complete-case population. Sixty-four patients (42.7%), i.e., all patients who completed the 12-month course and the 15-month visit, had documented values at each time point for every instrument, allowing comprehensive paired comparisons within subjects throughout the entire follow-up period.

Last observation carried forward (LOCF) population: for patients who discontinued treatment, the last value recorded before discontinuation was carried forward to all subsequent time points; patients lost to follow-up contributed only the assessments actually performed. Since it assumes no further change after discontinuation, this analysis is not conservative when patients stop due to lack of efficacy and may overestimate the benefit in that subgroup. It is presented only as a conventional sensitivity analysis and is supplemented by the scenarios described in Section 2.6. The number of patients contributing a pain assessment was 150, 128, 115, 115, 113, 110, 109, and 109 at baseline and at 1, 2, 3, 6, 9, 12, and 15 months, respectively.

**Figure 1:**
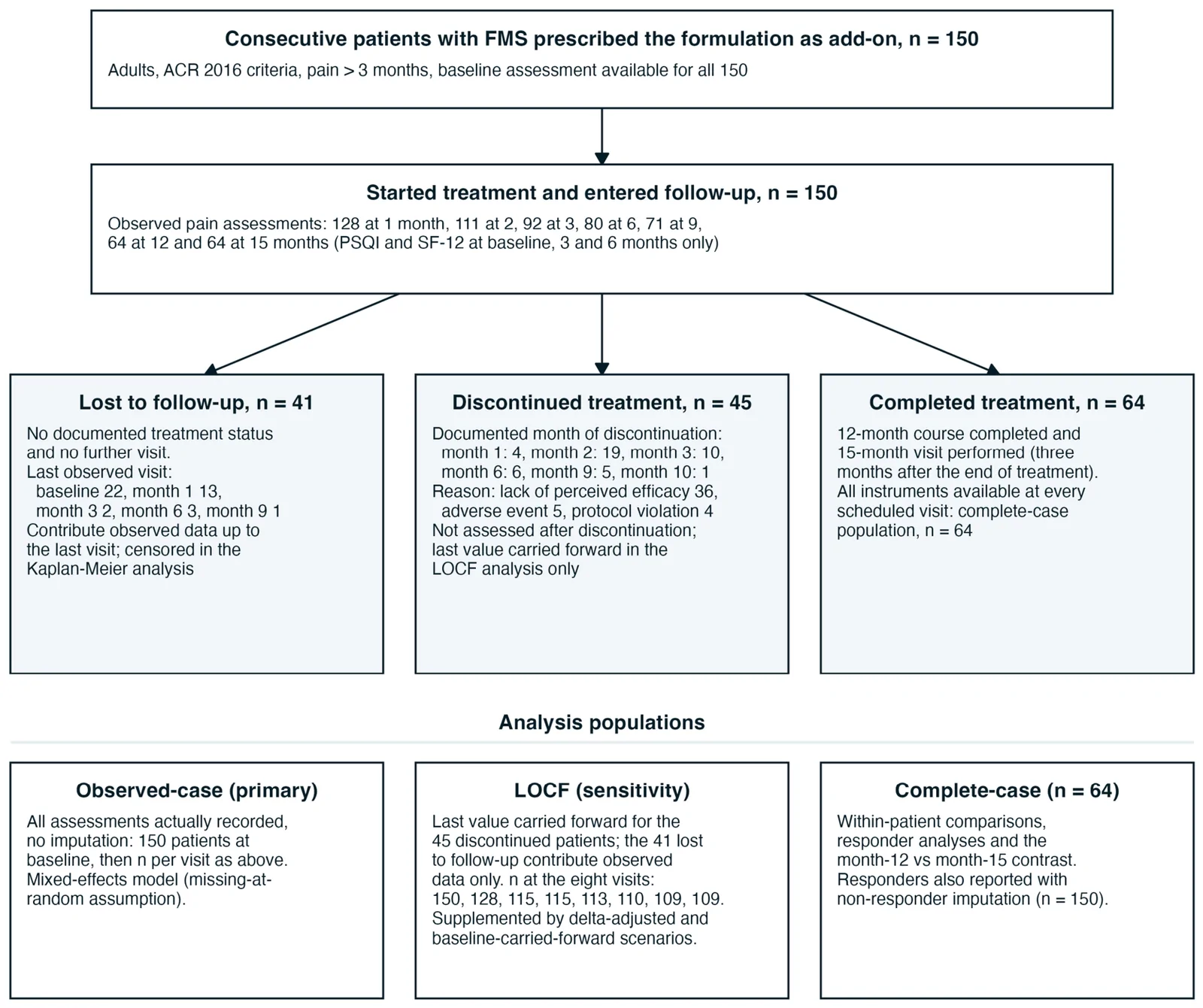
Flow of patients through the study and definition of the analysis populations. The numbers of observed pain assessments per visit are shown; the Pittsburgh Sleep Quality Index and the SF-12 were administered at baseline, 3 and 6 months only. LOCF, last observation carried forward.

Patients with no documented treatment status and no further visits were categorized as lost to follow-up (n = 41). Because the database was closed 15 months after the last enrollment, every scheduled visit had occurred for every patient; no patient was censored administratively, and every patient without a 15-month assessment is therefore either a documented discontinuation or lost to follow-up. Patients lost to follow-up contribute the assessments actually performed and are censored in the retention analysis. To identify attrition bias, the baseline characteristics of the complete-case population were compared with those of the other patients. The flow of patients and the analysis populations are summarized in Figure 1, and the proportion of missing values for each outcome at each visit is given in Table S1.

### 2.6. Statistical Analysis

Distributional assumptions were examined using the Shapiro–Wilk test. Baseline VAS, PSQI, SF-12 PCS, and SF-12 MCS departed significantly from normality (all *p* < 0.01); non-parametric methods were therefore used throughout, and continuous variables are summarised as the median and interquartile range (IQR). Categorical variables are summarised as absolute frequencies and percentages.

Within-subject changes from baseline were tested with the Wilcoxon signed-rank test with Holm correction for multiple comparisons, and the overall effect of time was determined using the Friedman test. The effect sizes for paired comparisons are reported as r = |Z|/sqrt(n).

In the observed-case population, the longitudinal course of each outcome was additionally modelled using linear mixed-effects models with a random intercept for patient, time point entered as a categorical fixed effect, and age as a covariate. This approach uses all available observations and is valid under a missing-at-random assumption. Estimated marginal means are reported with 95% confidence intervals (CI; Satterthwaite degrees of freedom); differences from baseline are reported with 95% CI and *p*-values adjusted for multiplicity by the Dunnett method.

Responder analyses followed the Initiative on Methods, Measurement, and Pain Assessment in Clinical Trials (IMMPACT) recommendations for interpreting the clinical importance of outcomes in chronic pain trials: a reduction in VAS of at least 30% from baseline was defined as a clinically meaningful response [33]. Additional responder thresholds were applied to secondary instruments. For the FIQR, no minimal clinically important difference has been derived in a peer-reviewed validation study; the 14% (8.1-point) reduction established for the original FIQ [34] was applied as a relative criterion, because the FIQR total score is scaled 0–100 like the FIQ, correlates strongly with it (r = 0.88) and has a similar mean value in the same patients [29]. The sensitivity of the responder proportions to this choice was examined with alternative FIQR thresholds (absolute reduction ≥ 8.1 points, relative reduction ≥ 20% and ≥30%). For the PSQI and the SF-12, no minimal clinically important difference has been validated in fibromyalgia; thresholds in common use in the chronic pain literature were therefore chosen before this dataset was analysed, namely a reduction of at least 3 points in the PSQI and an increase of at least 5 points in the SF-12 PCS and MCS. As the study is retrospective and no analysis plan was archived, all thresholds should be considered investigator-defined and not formally pre-specified. Responder proportions are reported for the complete-case population and, using non-responder imputation (where every patient without an observed assessment at that visit is counted as a non-responder), for all 150 patients who started treatment. Proportions were reported with 95% CI calculated using the Wilson score method.

Given that the primary reason for discontinuation was a perceived lack of efficacy, it is possible that outcomes are missing not at random (MNAR). Consequently, the assumption of missing-at-random in the mixed model may not be valid. The change from baseline in VAS and FIQR at 6 and 12 months was re-estimated under scenarios allowing unobserved outcomes to be worse than predicted: (i) LOCF; (ii) delta-adjustment, where the carried-forward value of each patient who discontinued due to inefficacy before the considered visit was worsened by δ = 5 to 30 points (VAS and FIQR units), while other discontinued patients retained their carried-forward value; (iii) baseline observation carried forward (BOCF) for all discontinued patients; and (iv) BOCF for all non-completers, including patients lost to follow-up, representing the worst case for all 150 patients who started treatment. The tipping point, i.e., the value of δ at which the mean change from baseline ceased to be statistically significant, is reported. The persistence of the effect after the end of treatment was tested directly by contrasting month 15 with month 12 in the mixed model and, in the complete-case population, by the Wilcoxon signed-rank test on the paired differences, with the mean paired difference and its 95% CI.

Retention on treatment during the 12-month treatment period was described using the Kaplan–Meier method, with treatment discontinuation for any stated reason as the event of interest and patients lost to follow-up censored at their last recorded visit; numbers at risk are reported, and, as a sensitivity analysis addressing the unknown treatment status of these patients, the analysis was repeated with patients lost to follow-up counted as discontinuations at their last visit. Baseline comparisons between populations used the Mann–Whitney U (Wilcoxon rank-sum) test and Fisher’s exact test. All tests were two-sided with a significance threshold of 0.05. Analyses were performed using R (version 4.3 or later; packages lme4, lmerTest, emmeans, survival, gtsummary, flextable, ggplot2).

### 2.7. Ethics

The study was conducted in accordance with the Declaration of Helsinki and approved by the Ethics Committee for Clinical Research of the Provinces of Verona and Rovigo (CESC; RED Register for Retrospective Studies, protocol code 1751CESC, approved on 23 May 2018). All patients had given written informed consent to the collection of their clinical data and to their use in anonymised form for research purposes, using the consent form of the RED Register approved by the same Committee. All data were anonymised prior to analysis; patients were identified by sequential study codes with no link to identifying information retained in the analysis dataset.

## 3. Results

### 3.1. Patients and Follow-Up

A total of 150 consecutive patients were included in the study. The cohort was predominantly female (131/150, 87.3%), with a median age of 51.0 years (IQR 43.0–59.0). Baseline symptom burden was high: median pain VAS was 80.0 (IQR 70.0–85.0) and 84 patients (56.0%) presented with severe pain (VAS ≥ 80). Median FIQR was 74.0 (IQR 65.0–84.0), median PSQI was 11.0 (IQR 9.0–12.0), and median SF-12 Physical and Mental Component Summary scores were 29.0 (IQR 25.0–33.0) and 26.0 (IQR 22.0–30.0), respectively, both far below the population norm of 50 [32] (Table 1).

**Table 1:** Baseline characteristics of the entire cohort (N = 150). Continuous variables are expressed as median [IQR], and categorical variables are expressed as n (%).

Characteristic	n	N = 150
Age, years, median [IQR]	150	51.0 [43.0–59.0]
Sex, n (%)	150	
F		131 (87.3%)
M		19 (12.7%)
Baseline pain severity, n (%)	150	
Moderate (VAS < 80)		66 (44.0%)
Severe (VAS ≥ 80)		84 (56.0%)
Pain VAS (0–100), median [IQR]	150	80.0 [70.0–85.0]
FIQR (0–100), median [IQR]	150	74.0 [65.0–84.0]
PSQI (0–21), median [IQR]	150	11.0 [9.0–12.0]
SF-12 PCS, median [IQR]	150	29.0 [25.0–33.0]
SF-12 MCS, median [IQR]	150	26.0 [22.0–30.0]
Follow-up status at 15 months, n (%)	150	
Completed 12-month treatment and 15-month observation		64 (42.7%)
Discontinued treatment		45 (30.0%)
Lost to follow-up		41 (27.3%)
Reason for discontinuation, n (%)	45	
Lack of perceived efficacy		36 (80.0%)
Adverse event		5 (11.1%)
Protocol violation		4 (8.9%)
Any adverse event, n (%)	150	15 (10.0%)
Treatment satisfaction (1–7), median [IQR]	115	5.0 [3.0–6.0]
Not recorded		35

n, number of patients with available variables. FIQR, revised Fibromyalgia Impact Questionnaire; PSQI, Pittsburgh Sleep Quality Index; SF-12 PCS/MCS, 12-item Short Form Health Survey Physical/Mental Component Summary; VAS, visual analogue scale; IQR, interquartile range; CI, confidence interval. A reason for discontinuation is recorded for all 45 patients who discontinued. Lost to follow-up includes one patient recorded as continuing treatment but not assessed after month 1. Baseline characteristics of the 64 patients with complete follow-up are given in Table 2. Any adverse event: 15/150 (10.0%, 95% CI 6.2–15.8). Treatment satisfaction was not recorded for 35 patients.

**Table 2:** Baseline characteristics according to completeness of follow-up.

Characteristic	Whole Cohort (N = 150)	Complete Follow-Up (n = 64)	Incomplete Follow-Up (n = 86)	*p*
Age, years	51.0 [43.0–59.0]	52.0 [45.5–58.5]	49.0 [42.0–59.0]	0.209
Sex				0.049
F	131 (87.3%)	60 (93.8%)	71 (82.6%)	
M	19 (12.7%)	4 (6.3%)	15 (17.4%)	
Baseline pain severity				0.509
Moderate (VAS < 80)	66 (44.0%)	26 (40.6%)	40 (46.5%)	
Severe (VAS ≥ 80)	84 (56.0%)	38 (59.4%)	46 (53.5%)	
Pain VAS (0–100)	80.0 [70.0–85.0]	80.0 [70.0–85.0]	80.0 [70.0–80.0]	0.451
FIQR (0–100)	74.0 [65.0–84.0]	76.0 [66.0–86.0]	72.0 [64.0–82.0]	0.269
PSQI (0–21)	11.0 [9.0–12.0]	11.0 [9.0–12.5]	11.0 [9.0–12.0]	0.341
SF-12 PCS	29.0 [25.0–33.0]	29.0 [25.0–34.0]	29.0 [25.0–32.0]	0.828
SF-12 MCS	26.0 [22.0–30.0]	25.0 [21.0–28.0]	26.5 [22.0–32.0]	0.129

Median [Q1–Q3] or n (%). *p*-values: Wilcoxon rank-sum test for continuous variables, Fisher exact test for categorical variables.

The flow of patients is shown in Figure 1. Sixty-four patients (42.7%) completed the 12-month treatment period and the observation at 15 months, 45 (30.0%) had a documented treatment discontinuation, and 41 (27.3%) were lost to follow-up without a documented treatment status; the latter group includes one patient recorded as continuing treatment who was not assessed after month 1. Among those who discontinued treatment, the stated reason was lack of perceived efficacy in 36/45, an adverse event in 5/45, and a protocol violation in 4/45; discontinuations occurred at months 1 (n = 4), 2 (19), 3 (10), 6 (6), 9 (5) and 10 (1). Patients lost to follow-up had their last visit at baseline (22), month 1 (13), month 3 (2), month 6 (3) or month 9 (1) (Table 1, Figure 1). The proportion of missing values per outcome and visit is reported in Table S1.

### 3.2. Completeness of Follow-Up and Attrition

The complete-case population comprised 64/150 patients (42.7%), i.e., all patients who completed treatment. Comparison with the remaining 86 patients showed no significant difference in any baseline clinical characteristic: age (52.0 [45.5–58.5] versus 49.0 [42.0–59.0], *p* = 0.209), pain VAS (80.0 [70.0–85.0] versus 80.0 [70.0–80.0], *p* = 0.451), FIQR (*p* = 0.269), PSQI (*p* = 0.341), SF-12 PCS (*p* = 0.828), SF-12 MCS (*p* = 0.129), or the proportion of patients with severe baseline pain (59.4% versus 53.5%, *p* = 0.509). The only difference reaching conventional significance was sex, with a higher proportion of women among patients with complete follow-up (93.8% versus 82.6%, *p* = 0.049) (Table 2).

Patients who continued under observation did not exhibit differences in disease severity at presentation compared to those who did not remain, suggesting that attrition was not influenced by the initial clinical status. However, this does not preclude attrition related to treatment response, as the most commonly cited reason for discontinuation was a perceived lack of efficacy.

### 3.3. Pain Intensity

Pain intensity decreased over the observation period (overall effect of time *p* < 0.001). In the mixed-effects model fitted to all available observations, the estimated mean VAS fell from 76.3 (95% CI 73.7–78.9) at baseline to 61.5 (58.8–64.2) at 1 month, 56.3 (53.4–59.2) at 3 months, 55.0 (52.0–58.0) at 6 months, 52.3 (49.1–55.4) at 12 months and 52.8 (49.6–56.0) at 15 months. Estimated mean differences from baseline were −14.8 points (95% CI −17.5 to −12.0) at 1 month, −20.0 (−23.1 to −16.9) at 3 months, −21.3 (−24.6 to −18.0) at 6 months, −24.1 (−27.6 to −20.5) at 12 months and −23.5 (−27.1 to −20.0) at 15 months, all *p* < 0.001 after adjustment for multiplicity (Dunnett-adjusted CI; Table 3).

**Figure 2:**
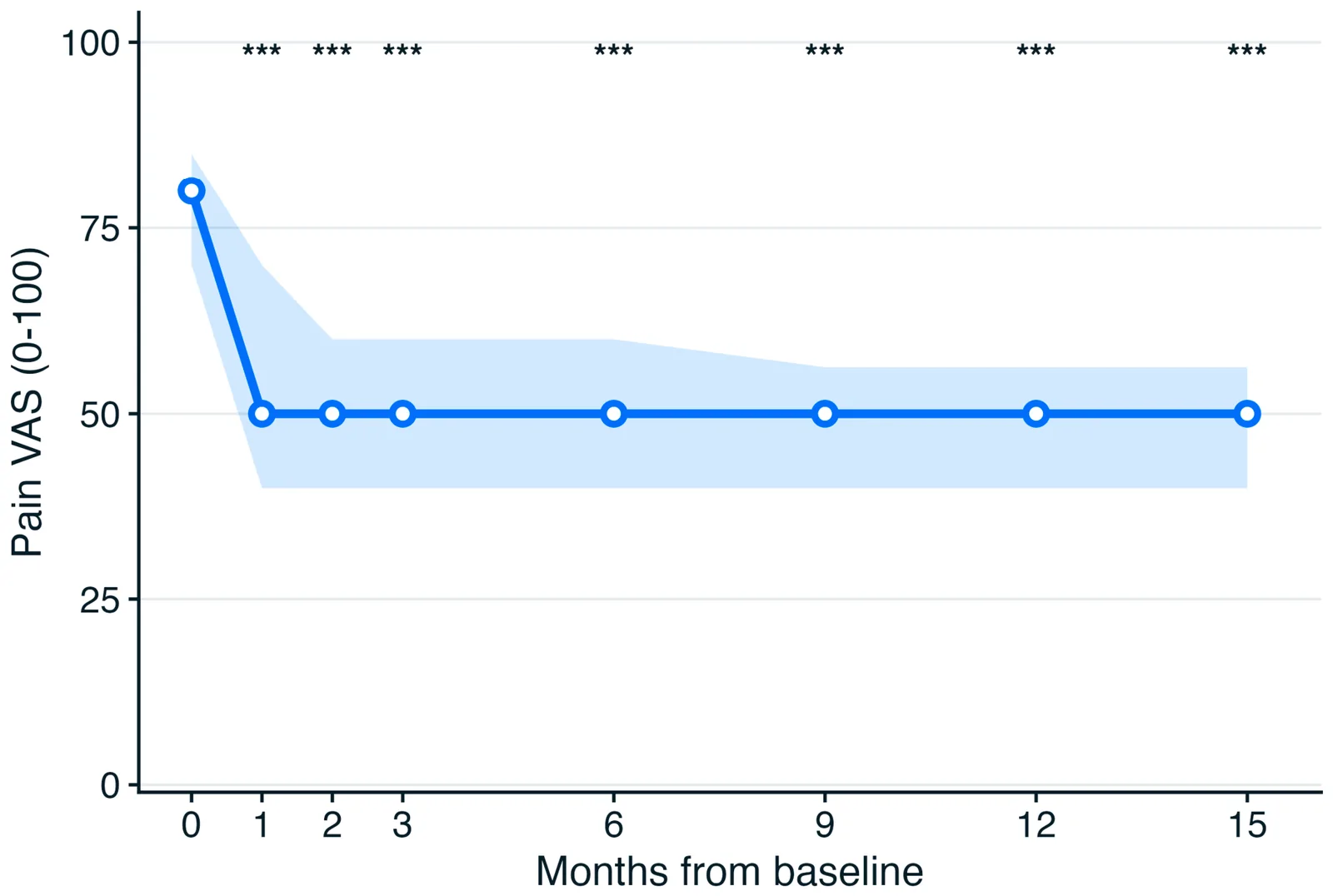
Course of pain intensity over 15 months in the complete-case population (n = 64). Line, median; band, interquartile range (IQR). Asterisks denote paired comparisons versus baseline (Wilcoxon signed-rank test, Holm-adjusted); *** *p* < 0.001. The subtitle reports the proportion of patients who reached the IMMPACT 30% pain-reduction threshold at 6 months.

The largest single change occurred within the first month and accounted for approximately two-thirds of the total change observed over 15 months. Thereafter, the reduction continued at a slower rate, and no rebound towards baseline values was apparent at any point among patients who remained under observation (Figure 2). At 15 months, three months after the end of treatment, the estimated mean VAS was 52.8 versus 52.3 at 12 months (difference +0.5 points, 95% CI −2.4 to +3.5, *p* = 0.72), and the FIQR 56.8 versus 56.1 (+0.7, −1.9 to +3.3, *p* = 0.59) (Table 3). This contrast is informative only for the 64 patients who completed the course and attended both visits: among them, VAS was identical at the two visits in 54 patients, higher at 15 months in 8, and lower in 2 (mean paired difference +0.5 points, 95% CI +0.0 to +1.1; Wilcoxon *p* = 0.059). FIQR was identical in 43 patients, higher in 16, and lower in 5 (mean +0.7, 95% CI +0.3 to +1.2; *p* = 0.004), indicating a statistically detectable but clinically negligible increase of less than one point. Patients who discontinued earlier were not re-assessed at 15 months, so no inference about persistence is possible for them. In the complete-case population, the median VAS decreased from 80.0 (IQR 70.0–85.0) to 50.0 (IQR 40.0–57.5) at 15 months, a median within-patient change of −30.0 points (IQR −40.0 to −20.0; *p* < 0.001, r = 0.87) (Table 4 and Table 5).

**Table 4:** Outcome measures over 15 months in the complete-case population (n = 64). Median [IQR].

Outcome	Baseline	1 Mo	2 Mo	3 Mo	6 Mo	9 Mo	12 Mo	15 Mo
Pain VAS (0–100)	80.0 [70.0–85.0]	50.0 [40.0–70.0]	50.0 [40.0–60.0]	50.0 [40.0–60.0]	50.0 [40.0–60.0]	50.0 [40.0–57.5]	50.0 [40.0–57.5]	50.0 [40.0–57.5]
FIQR (0–100)	76.0 [66.0–86.0]	59.0 [45.0–70.0]	55.0 [45.0–64.0]	55.0 [45.0–61.0]	55.0 [45.0–61.5]	55.0 [45.0–60.5]	55.0 [45.0–61.5]	55.0 [45.0–61.5]
PSQI (0–21)	11.0 [9.0–12.5]	—	—	10.0 [7.5–12.0]	10.0 [7.5–11.0]	—	—	—
SF-12 PCS	29.0 [25.0–34.0]	—	—	33.0 [26.7–40.0]	33.5 [28.5–40.0]	—	—	—
SF-12 MCS	25.0 [21.0–28.0]	—	—	29.0 [24.0–34.0]	30.5 [25.0–35.0]	—	—	—

PSQI, SF-12 PCS, and SF-12 MCS were administered at baseline, 3 and 6 months only; dashes indicate timepoints at which the instrument was not administered.

**Table 5:** Within-patient comparisons versus baseline in the complete-case population (n = 64).

Month	n	Median [IQR]	Median Change	*p* (Holm)	Effect Size r	Signif.
Pain VAS						
1	64	50.0 [40.0–70.0]	−20.0	<0.001	0.82	***
2	64	50.0 [40.0–60.0]	−20.0	<0.001	0.85	***
3	64	50.0 [40.0–60.0]	−25.0	<0.001	0.86	***
6	64	50.0 [40.0–60.0]	−25.0	<0.001	0.87	***
9	64	50.0 [40.0–57.5]	−30.0	<0.001	0.87	***
12	64	50.0 [40.0–57.5]	−30.0	<0.001	0.87	***
15	64	50.0 [40.0–57.5]	−30.0	<0.001	0.87	***
FIQR						
1	64	59.0 [45.0–70.0]	−14.6	<0.001	0.80	***
2	64	55.0 [45.0–64.0]	−19.0	<0.001	0.83	***
3	64	55.0 [45.0–61.0]	−21.5	<0.001	0.85	***
6	64	55.0 [45.0–61.5]	−21.5	<0.001	0.85	***
9	64	55.0 [45.0–60.5]	−22.0	<0.001	0.85	***
12	64	55.0 [45.0–61.5]	−23.0	<0.001	0.86	***
15	64	55.0 [45.0–61.5]	−20.5	<0.001	0.86	***
PSQI						
3	64	10.0 [7.5–12.0]	−1.0	<0.001	0.53	***
6	64	10.0 [7.5–11.0]	−1.0	<0.001	0.54	***
SF-12 PCS						
3	64	33.0 [26.7–40.0]	+3.5	<0.001	0.75	***
6	64	33.5 [28.5–40.0]	+5.0	<0.001	0.79	***
SF-12 MCS						
3	64	29.0 [24.0–34.0]	+3.0	<0.001	0.74	***
6	64	30.5 [25.0–35.0]	+3.0	<0.001	0.77	***

Wilcoxon signed-rank test; *p*-values adjusted using the Holm method. Effect size r = |Z|/sqrt(n). Asterisks: *** *p* < 0.001; Each row compares every patient with his or her own baseline value in the same 64 patients: n is the number of paired observations, Median [IQR] is the score recorded at that visit, and Median change is the median of the individual differences from baseline, which need not equal the difference between the two medians. The effect size r expresses the magnitude of the within-patient shift on a 0–1 scale, conventionally interpreted as small around 0.10, medium around 0.30, and large from 0.50 upwards. Pain and disease impact show large effects that are established by month 1 and maintained to month 15; both SF-12 components, measured only at baseline, 3 and 6 months, show large effects at those two visits; sleep reaches the same statistical significance with about half the effect size and a median change of one PSQI point; the clinical relevance of these magnitudes is assessed in Table 6 and Table 7.

**Table 6:** Patients who reached clinically meaningful thresholds, in the complete-case population (n = 64) and in all patients who started treatment, with non-responder imputation (n = 150).

Criterion	Month	Responders	% [95% CI]
Completers (n = 64)			
FIQR reduction ≥ 14%	6	50/64	78.1 [66.6–86.5]
VAS reduction ≥ 30%	6	38/64	59.4 [47.1–70.5]
SF-12 PCS increase ≥ 5	6	34/64	53.1 [41.1–64.8]
SF-12 MCS increase ≥ 5	6	24/64	37.5 [26.7–49.7]
PSQI reduction ≥ 3 points	6	13/64	20.3 [12.3–31.7]
VAS reduction ≥ 30%	15	41/64	64.1 [51.8–74.7]
FIQR reduction ≥ 14%	15	48/64	75.0 [63.2–84.0]
All patients who started treatment, non-responder imputation (n = 150)			
FIQR reduction ≥ 14%	6	56/150	37.3 [30.0–45.3]
VAS reduction ≥ 30%	6	43/150	28.7 [22.0–36.4]
SF-12 PCS increase ≥ 5	6	35/150	23.3 [17.3–30.7]
SF-12 MCS increase ≥ 5	6	26/150	17.3 [12.1–24.2]
PSQI reduction ≥ 3 points	6	15/150	10.0 [6.2–15.8]
VAS reduction ≥ 30%	15	41/150	27.3 [20.8–35.0]
FIQR reduction ≥ 14%	15	48/150	32.0 [25.1–39.8]

Thresholds: IMMPACT criterion for VAS [33]; 14% reduction for the FIQR, derived from the original FIQ [34]; investigator-defined thresholds for PSQI and SF-12, for which no validated minimal clinically important difference is available in fibromyalgia. Non-responder imputation: every patient without an observed assessment at that visit (discontinued or lost to follow-up) is counted as a non-responder. 95% CI using the Wilson method.

**Table 7:** Sensitivity of the mean change from baseline at 12 months to assumptions about missing data (VAS and FIQR, 0–100).

Scenario	n	VAS, Change at 12 Months [95% CI]	FIQR, Change at 12 Months [95% CI]
Mixed-effects model, observed cases (missing at random; Table 3)	150 (64 at 12 months)	−24.1 [−27.6, −20.5]	−17.5 [−20.6, −14.4]
Complete cases only	64	−30.5 [−34.0, −27.1]	−22.1 [−25.9, −18.4]
LOCF for discontinued patients	109	−19.4 [−23.1, −15.8]	−13.2 [−16.4, −10.1]
Delta-adjusted LOCF, δ = 10 (inefficacy discontinuations)	109	−16.6 [−20.8, −12.4]	−10.0 [−13.7, −6.2]
Delta-adjusted LOCF, δ = 20	109	−14.3 [−19.0, −9.6]	−7.1 [−11.5, −2.7] (*p* = 0.004)
Delta-adjusted LOCF, δ = 30	109	−12.8 [−17.9, −7.8] (*p* < 0.001)	−4.9 [−9.8, 0.1] (*p* = 0.109)
BOCF for all discontinued patients	109	−17.9 [−21.4, −14.4]	−13.0 [−16.0, −10.0]
BOCF for all non-completers (worst case)	150	−13.0 [−15.9, −10.2]	−9.4 [−11.8, −7.1]

Mean change from baseline with 95% CI (t-based; Dunnett-adjusted for the mixed model, as in Table 3); all changes *p* < 0.001 (Wilcoxon signed-rank) unless stated. LOCF, last observation carried forward (the last value recorded before discontinuation); δ, number of points added to the carried-forward value of patients who discontinued for lack of perceived efficacy (n = 36); BOCF, baseline observation carried forward (the patient is assumed to have returned to the baseline value). Patients lost to follow-up contribute only in the mixed model (observed visits) and in the worst-case scenario. Results at 6 months are given in Table S2.

### 3.4. Disease Impact

**Figure 3:**
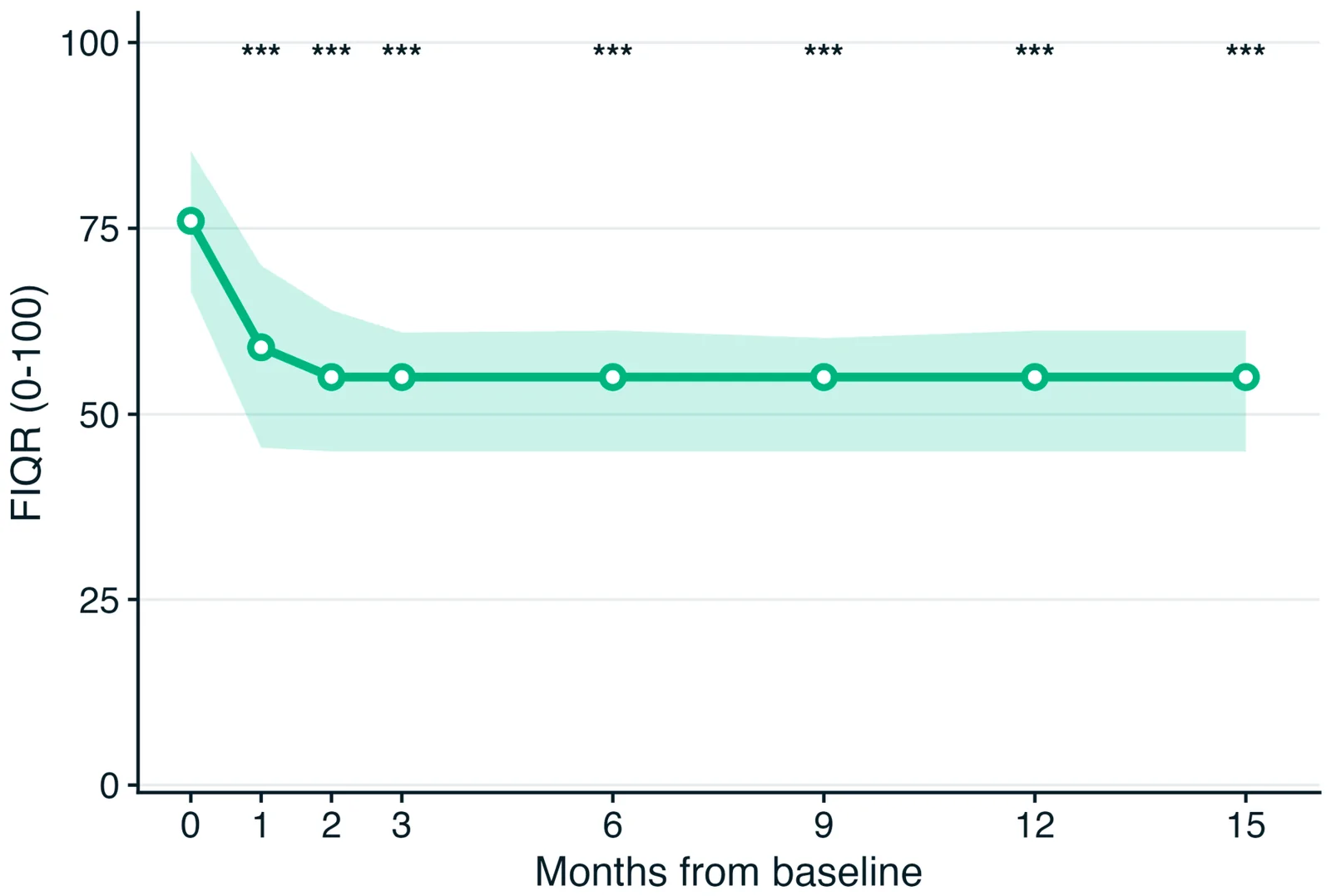
Course of disease impact (revised Fibromyalgia Impact Questionnaire) over 15 months in the complete-case population (n = 64). Line, median; band, interquartile range; asterisks as in Figure 2.

Disease impact followed a comparable pattern. The estimated mean FIQR decreased from 73.6 (95% CI 71.5–75.8) at baseline to 58.3 (55.8–60.7) at 3 months, 57.3 (54.8–59.8) at 6 months, 56.1 (53.5–58.8) at 12 months and 56.8 (54.2–59.5) at 15 months, with mean differences from baseline of −15.4 (−18.1 to −12.6), −16.3 (−19.2 to −13.5), −17.5 (−20.6 to −14.4) and −16.8 (−19.9 to −13.7) points, respectively (all *p* < 0.001) (Table 3). In the complete-case population, the median FIQR fell from 76.0 (66.0–86.0) to 55.0 (45.0–61.5) at 15 months, a median within-patient change of −20.5 points (IQR −29.5 to −9.0; *p* < 0.001, r = 0.86) (Table 4 and Table 5, Figure 3).

### 3.5. Sleep Quality

**Figure 4:**
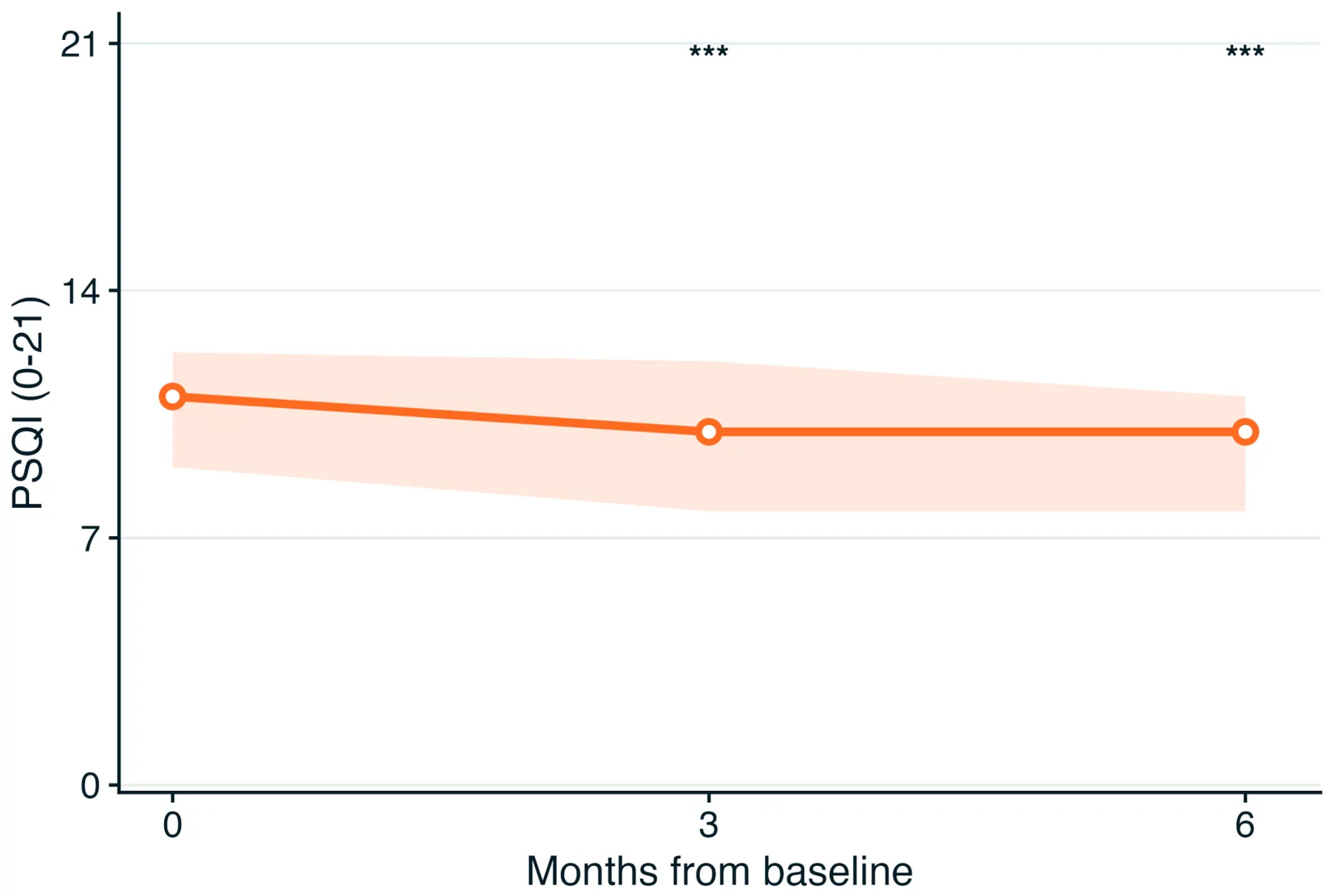
Course of sleep quality (Pittsburgh Sleep Quality Index) in the complete-case population (n = 64). The scale is displayed over its full 0–21 range; the change of one point is statistically significant but well below the 3-point threshold for clinical relevance, which only 20.3% of patients reached. Asterisks as in Figure 2.

**Figure 5:**
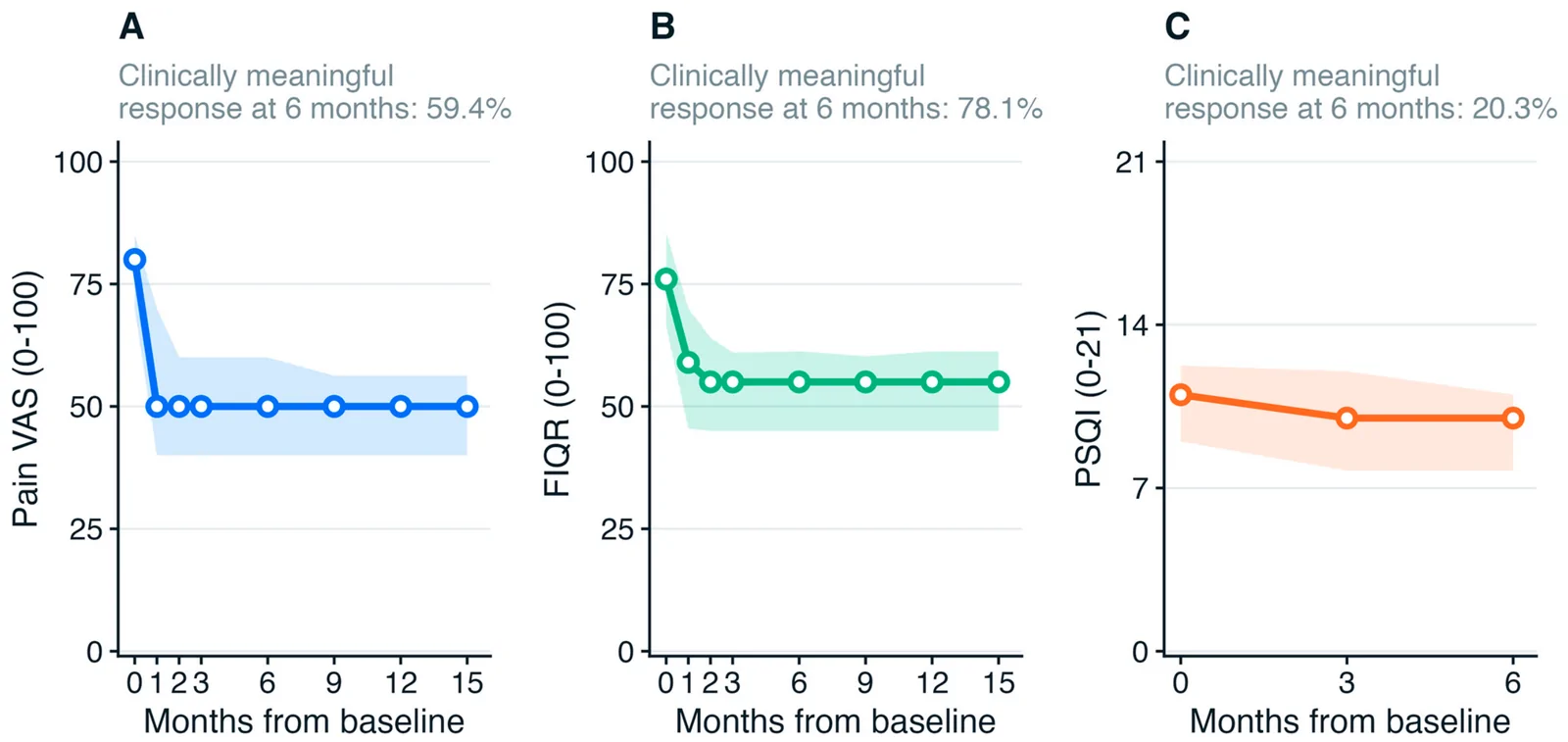
The three outcome domains side by side in the complete-case population (n = 64): (**A**) pain VAS; (**B**) FIQR; and (**C**) PSQI. Each panel is drawn over the full range of its scale. All time points of the three scales differed significantly from baseline (Wilcoxon signed-rank, Holm-adjusted); however, the proportion of patients reaching a clinically meaningful improvement, reported in each subtitle, ranged from 78.1% for disease impact to 20.3% for sleep.

Sleep quality changed considerably less than in any other domain. The estimated mean PSQI decreased from 10.6 (95% CI 10.1–11.0) at baseline to 9.7 (9.3–10.2) at 3 months and 9.6 (9.1–10.1) at 6 months, corresponding to mean differences of −0.8 (−1.2 to −0.5) and −1.0 (−1.3 to −0.6) points. Although these changes reached statistical significance (*p* < 0.001), their magnitude was well below the 3-point threshold for a clinically meaningful improvement, and only 20.3% of complete cases reached that threshold at 6 months. Median PSQI moved from 11.0 (IQR 9.0–12.5) to 10.0 (IQR 7.5–11.0), leaving the majority of patients within the range indicating poor sleep quality [31] (Table 3, Table 4 and Table 5, Figure 4 and Figure 5C).

This dissociation between pain, physical function, mental health, and sleep was consistent across all analyses, populations, and methods of handling incomplete follow-up, within the six-month window in which PSQI and SF-12 were measured; it could not be examined at later visits.

### 3.6. Health-Related Quality of Life

Both SF-12 component scores improved. The estimated mean PCS rose from 29.2 (95% CI 27.9–30.4) at baseline to 32.8 (31.5–34.2) at 3 months and 34.0 (32.6–35.4) at 6 months, a mean difference of +4.8 points (+3.8 to +5.8; *p* < 0.001). The estimated mean MCS rose from 26.6 (25.3–27.9) to 29.7 (28.3–31.1) and 30.5 (29.0–31.9), a mean difference of +3.9 points (+2.8 to +4.9; *p* < 0.001) (Table 3). At 6 months, 53.1% of complete cases had gained at least 5 PCS points and 37.5% at least 5 MCS points (Table 4, Table 5 and Table 6).

### 3.7. Responder Analysis

Responder proportions are reported in two ways (Table 6): among the 64 patients who completed treatment, in whom they describe the response of patients who stayed on the formulation, and among all 150 patients who started treatment with non-responder imputation, in which every patient without an observed assessment at that visit (discontinued or lost) is counted as a non-responder; the latter is necessarily lower and bounds the proportion from below. Using the IMMPACT criterion of at least a 30% reduction in pain VAS [33], 59.4% of completers were classified as responders at 6 months (95% CI 47.1–70.5) and 64.1% at 15 months (51.8–74.7); with non-responder imputation, the corresponding proportions were 28.7% (43/150, 95% CI 22.0–36.4) and 27.3% (41/150, 20.8–35.0).

**Figure 6:**
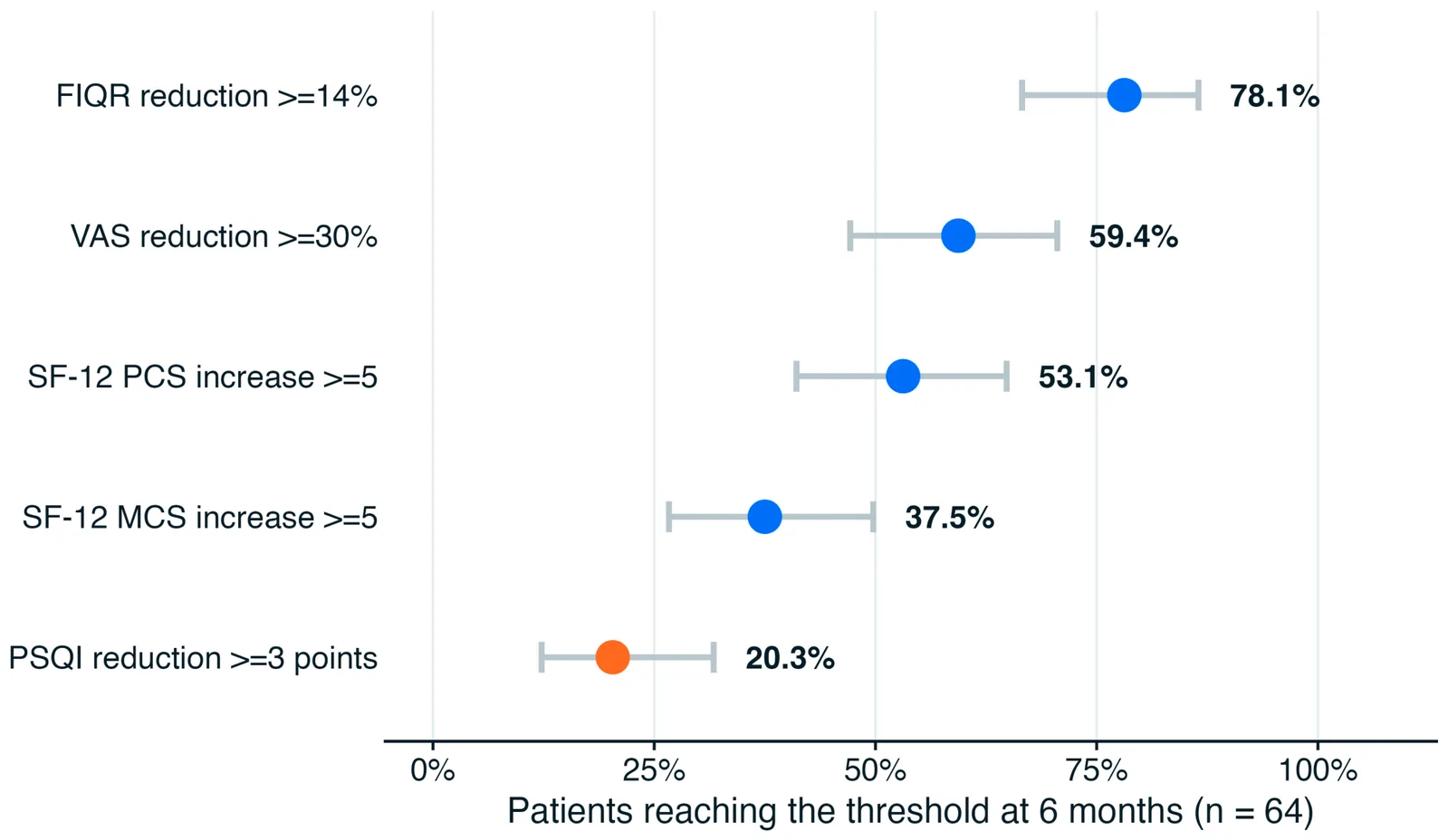
Proportion of patients reaching clinically meaningful thresholds at 6 months (complete-case population, n = 64). Horizontal bars indicate 95% CI (Wilson method). Sleep, in orange, was the only domain in which fewer than one patient in four improved meaningfully.

Applying the 14% FIQR threshold [34], 78.1% of completers reached the criterion at 6 months and 75.0% at 15 months, corresponding to 37.3% (56/150) and 32.0% (48/150) of all patients who started. The proportion of completers reaching the criterion at 6 months was 81.2% with an absolute threshold of 8.1 points, 67.2% with a 20% threshold and 42.2% with a 30% threshold (39.3%, 32.7% and 20.0% of all 150 patients). The corresponding proportions for a PSQI reduction of at least 3 points at 6 months were 20.3% of completers and 10.0% of all patients (Figure 6, Table 6). The heterogeneity of individual trajectories underlying these group figures is shown in Figure S1.

### 3.8. Retention on Treatment

**Figure 7:**
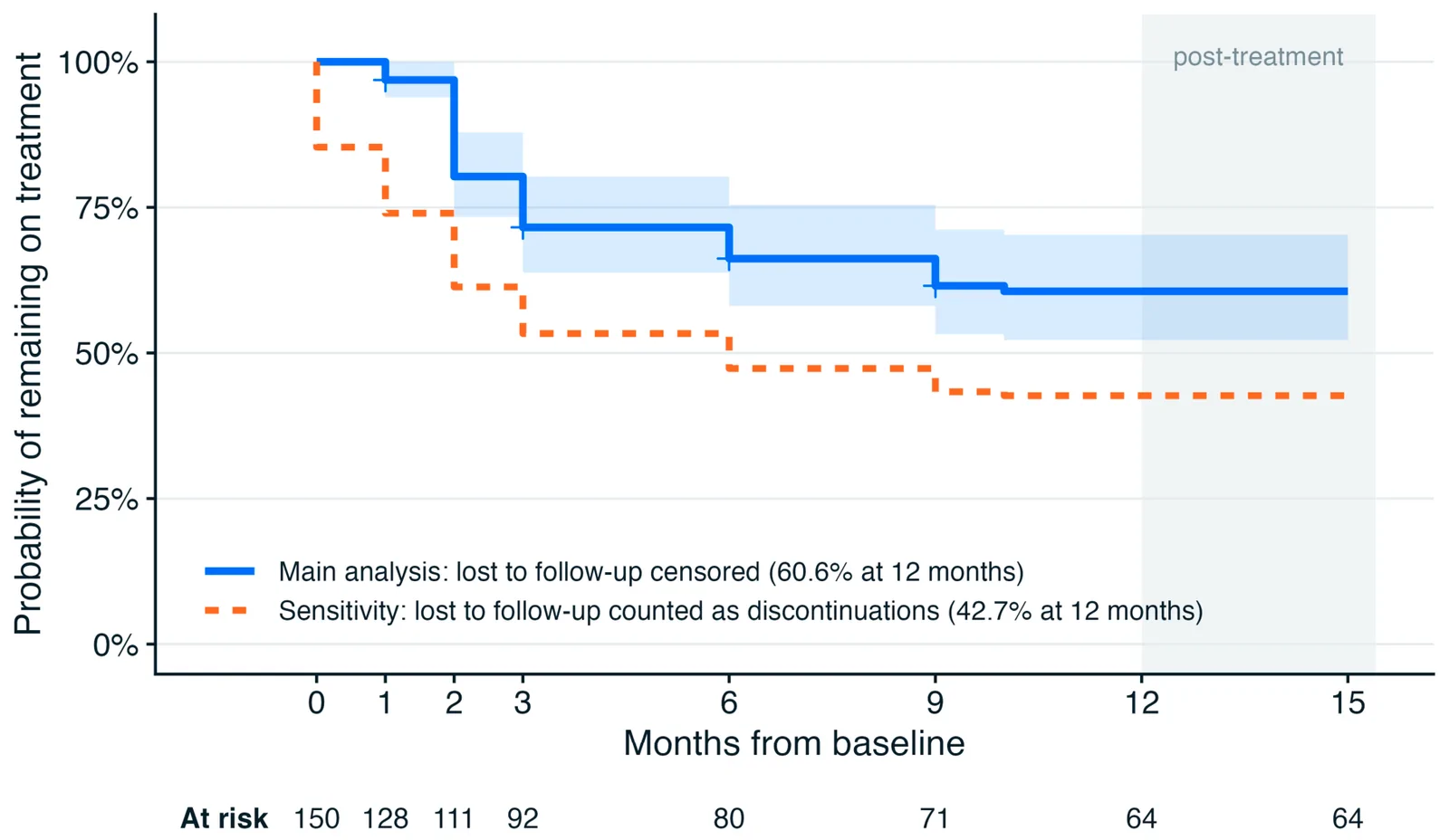
Kaplan–Meier estimate of the probability of remaining on treatment (N = 150). The event is documented discontinuation for any stated reason (45 events, none after month 10). Main analysis (solid line, 95% CI band): patients lost to follow-up are censored at their last observed visit (ticks); patients who completed the 12-month course are censored at 15 months. Sensitivity analysis (dashed line): the 41 patients lost to follow-up are counted as discontinuations at their last observed visit. Numbers at risk are shown below the plot. Treatment was scheduled for 12 months; the shaded area marks the post-treatment period.

The estimated probability of remaining on treatment was 71.6% (95% CI 63.8–80.3) at 3 months, 66.2% (58.1–75.4) at 6 months, and 60.6% (52.2–70.3) at 12 months, marking the end of the treatment period (Figure 7, which also reports the numbers at risk). Discontinuations were concentrated early, with 33 of the 45 events occurring within the first 3 months, and none recorded after month 10. Because the treatment status of the 41 patients lost to follow-up is unknown, the analysis was repeated, counting them as discontinuations at their last visit: under this lower-bound assumption, the probability of remaining on treatment was 53.3% (45.9–61.9) at 3 months, 47.3% (40.0–56.0) at 6 months, and 42.7% (35.4–51.4) at 12 months. The true retention lies between the two estimates.

### 3.9. Safety and Treatment Satisfaction

Fifteen patients (10.0%, 95% CI 6.2–15.8) had at least one adverse event recorded; adverse events were ascertained passively (spontaneous report or physician record), and no event could be recorded after a patient was lost to follow-up. The most frequent adverse events were insomnia (n = 9, alone in 6 and in combination in 3), nausea (n = 4, alone or in combination), headache (n = 3), dizziness (n = 2, always in combination), and hyperexcitability or tremor (n = 2). No serious adverse event was recorded among the events documented. Five patients (3.3% of the cohort) discontinued treatment because of an adverse event. Median treatment satisfaction on the 7-point scale was 5.0 (IQR 3.0–6.0) in the whole cohort and 5.0 (IQR 5.0–6.0) among patients with complete follow-up (Table 1).

### 3.10. Sensitivity Analyses

**Figure 8:**
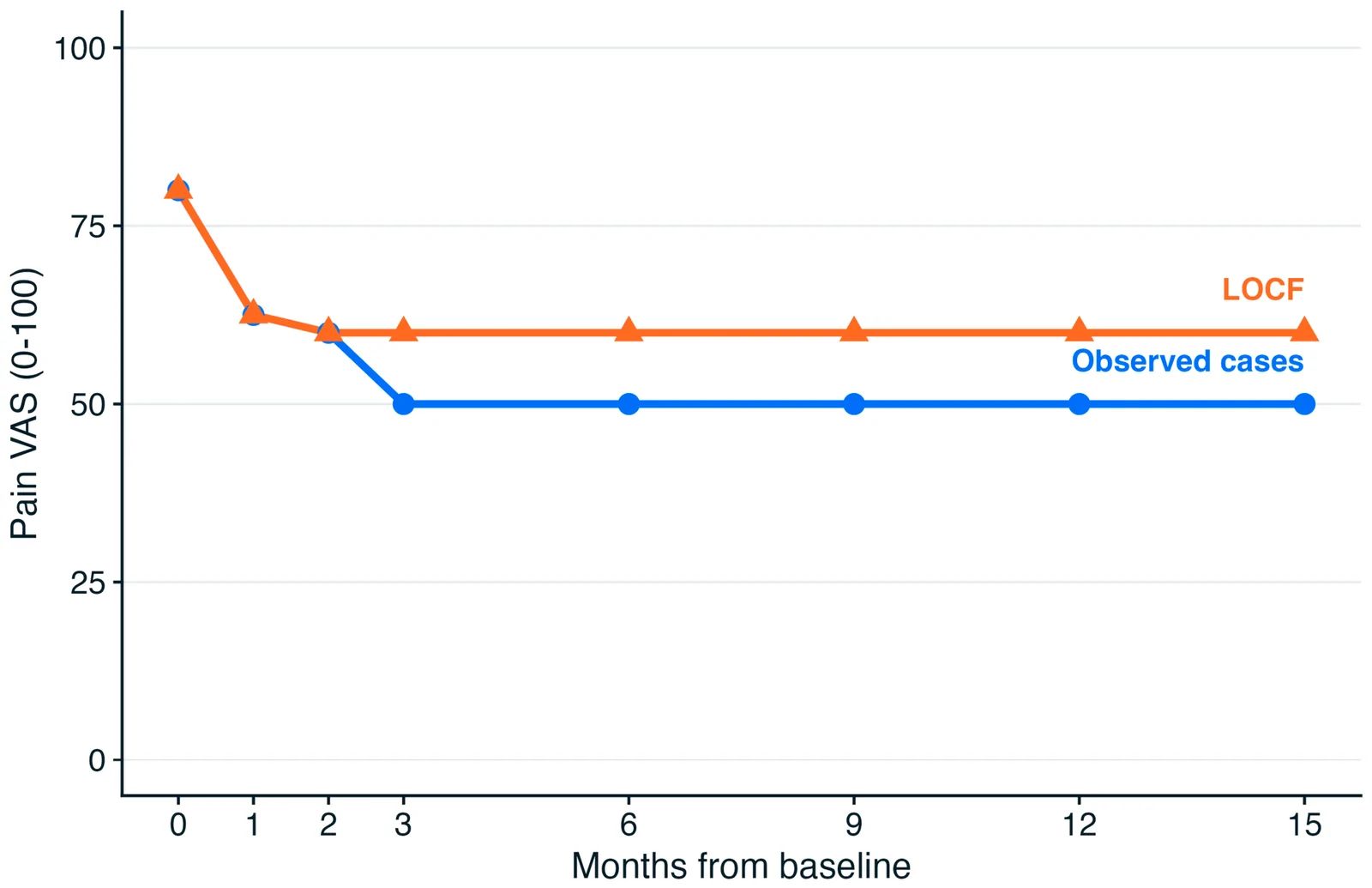
Median pain VAS in the whole cohort according to the handling of incomplete follow-up: observed cases versus LOCF. The divergence widens over time and reflects imputation rather than the treatment effect.

**Figure 9:**
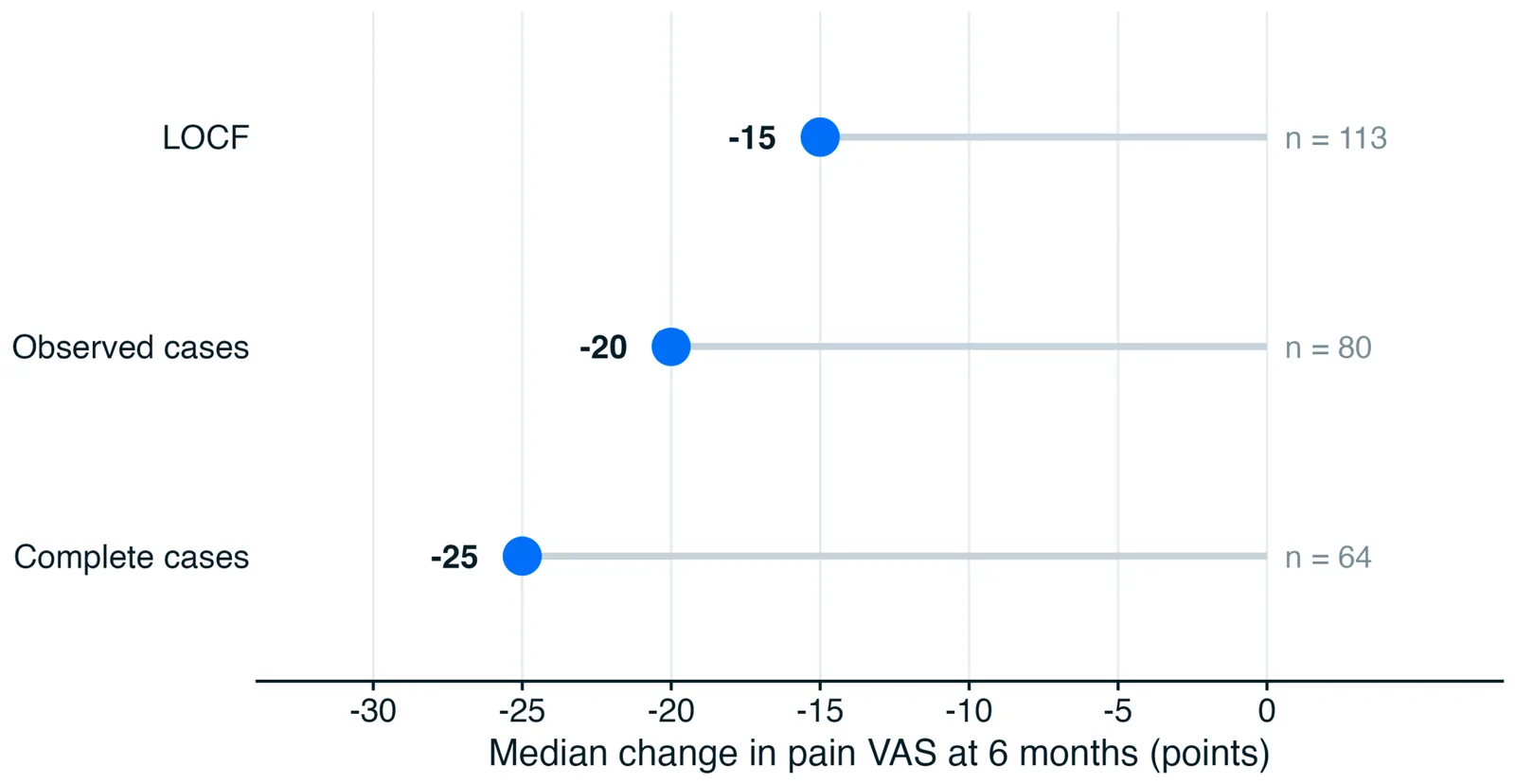
Median change in pain VAS at 6 months according to the analysis population. The same outcome at the same timepoint yields −15, −20, or −25 points depending on the denominator; the gradient reflects attrition.

The magnitude of the observed change depended systematically on how incomplete follow-up was handled (Figure 8). The median VAS change from baseline at 6 months was −15.0 points under the LOCF analysis, −20.0 among all patients with an observed assessment, and −25.0 among complete cases (Figure 9). At 15 months, the divergence between the observed and LOCF trajectories reached 10 VAS points (Figure 8). The direction of the change was the same in every analysis, but its estimated size differed by up to a third depending on the population analysed.

Table 7 presents the mean change from baseline at 12 months, assuming that the unobserved outcomes of patients who discontinued treatment are worse than predicted. For VAS, the change was −24.1 points in the mixed model, −19.4 (95% CI −23.1 to −15.8) under LOCF (n = 109), −16.6 (95% CI −20.8 to −12.4) when the carried-forward value of patients who discontinued due to inefficacy was worsened by 10 points, −12.8 (95% CI −17.9 to −7.8) with δ = 30, −17.9 (95% CI −21.4 to −14.4) when all discontinued patients were assumed to have returned to baseline, and −13.0 (95% CI −15.9 to −10.2) when all 86 non-completers, including those lost to follow-up, were assumed to have returned to baseline (n = 150). For the FIQR, the corresponding changes were −17.5 (mixed model), −13.2 (95% CI −16.4 to −10.1) (LOCF), −10.0 (95% CI −13.7 to −6.2) (δ = 10), −7.1 (95% CI −11.5 to −2.7) (δ = 20, *p* = 0.004), −4.9 (95% CI −9.8 to 0.1) (δ = 30, *p* = 0.109), −13.0 (95% CI −16.0 to −10.0) (BOCF, discontinued), and −9.4 (95% CI −11.8 to −7.1) (BOCF, all non-completers). The change in VAS remained statistically significant under every scenario, whereas for the FIQR, the tipping point was reached at δ = 30, i.e., when patients who stopped due to inefficacy were assumed to have worsened by 30 points below their last recorded value. At 6 months, the change remained statistically significant under every scenario for both outcomes, with the FIQR tipping point being reached only at 12 months (Table S2). Under the worst-case scenario, the mean 12-month change among all patients who started treatment was about half of that estimated in the mixed model, and the responder proportions with non-responder imputation (Table 6) describe the same lower bound.

## 4. Discussion

### 4.1. Principal Findings

In a cohort of 150 consecutive patients with FMS treated in routine practice with a fixed multicomponent formulation containing PEA and ALC as an adjunct to stable standard care, reductions in pain intensity and disease impact were observed over a follow-up period of up to 15 months in the patients who remained under observation. Treatment was scheduled for 12 months, and the assessment at 15 months, three months after discontinuation, was intended to establish whether the analgesic effect persisted after withdrawal, i.e., whether the combination produced a long-lasting pain relief, as would be expected from the epigenetic mechanism proposed for ALC, in which acetylation-dependent up-regulation of mGlu2 receptors produces effects that outlast drug administration [35,36] (Section 4.4). Among the patients who completed the course, the improvement was sustained without rebound (Table 3 and Table 4, Figure 2 and Figure 3); nothing is known about the subsequent course of the patients who stopped treatment or were lost to follow-up. The majority of the improvement occurred within the first month, consistent with the time course reported for micron-size PEA in a meta-analysis of extended treatment [37]. Subsequently, the trajectory of improvement plateaued but did not reverse. The magnitude of pain reduction (estimated mean VAS from 76.3 to 52.8) is closely comparable to that reported at our centre for ultramicronised PEA alone (VAS from 75.84 to 52.49 over 15 months) and for ALC alone (VAS from 75.9 to 51.9 over 14 months) [17,18]. The three series come from different registries at the same centre and involve different patients, with no patient appearing in more than one series. Thus, the comparison is between independent samples from the same centre, based only on published summary statistics. Among completers, 78.1% reached the minimal clinically important difference for disease impact at six months and 59.4% met the IMMPACT threshold for pain relief [33,34]; when every patient who started treatment is counted and non-completers are classified as non-responders, these proportions fall to 37.3% and 28.7% (Table 6, Figure 6). The true proportion lies between these bounds and cannot be located more precisely with the available data. No serious adverse events were recorded during the 12-month treatment period, and 3.3% of patients discontinued due to a documented adverse event. However, adverse events were identified from clinical records rather than through systematic questioning (Section 4.5). An estimated 60.6% of patients remained on treatment throughout the 12-month period when patients lost to follow-up are censored, and 42.7% when they are counted as discontinuations (Figure 7). Among completers, pain and disease impact three months after the end of treatment were within one point of the values at 12 months. Over the six months in which it was measured, sleep improvement was statistically significant but clinically negligible (Figure 4 and Figure 5).

These results can be set against standard pharmacotherapy benchmarks, with the caveat that follows. The Cochrane overview shows that duloxetine, milnacipran, and pregabalin significantly relieved pain in about one in ten patients compared to a placebo, with no proven effectiveness beyond six months [13,14,15]. This group is not controlled, so the comparison is indirect. The size of the change seen cannot be turned into a treatment effect. The only valid conclusion is that the changes observed are large enough to be clinically relevant if they were confirmed against a comparator over a comparable period.

### 4.2. Comparison with the Randomised and Meta-Analytic Evidence

The sole randomised study investigating the combination therapy in FMS incorporated PEA 600 mg and ALC 500 mg, administered twice daily for 12 weeks to 142 patients who had already been stabilised on duloxetine and pregabalin for 12 weeks. This study demonstrated significant additional improvements in the Widespread Pain Index, the FIQR, and the modified Fibromyalgia Assessment Status [26]. In that trial, the combination enhanced the effect of conventional treatment. The present cohort does not replicate that finding, as it lacks a comparator; instead, it provides a description of what occurs over 15 months in routine practice, including the proportion of patients who discontinue treatment and when. The two designs address distinct questions, and the retention data presented here offer information that a 12-week randomized trial with protocol-mandated follow-up does not. The same group later reported a retrospective analysis of 77 patients from the Italian Fibromyalgia Registry treated with PEA and ALC for 24 months in addition to usual care: 28.6% achieved a reduction of at least 30% in the FIQR, FASmod, and polysymptomatic distress scores, 20.8% improved to a lesser extent, and 50.6% did not improve. Only patients who attended both the 12- and 24-month assessments were analyzed, so attrition could not be described, and pain intensity and sleep were not measured [27]. The proportion of markedly improved patients in that series is similar to the proportion of all patients who started treatment here and achieved a 30% reduction in FIQR at 6 months (20.0%). The present cohort adds data on pain intensity, sleep, quality of life, the timing and reasons for discontinuation, and a post-treatment visit. Regarding PEA as a monotherapy in chronic pain, three meta-analyses of randomised trials have reported significant reductions in pain intensity, improved quality of life, and a lack of major adverse effects [38,39,40]. Furthermore, the therapeutic effect has been observed to increase between the first and second month of treatment [37]. For ALC, a placebo-controlled trial revealed significant between-group differences at week 10 in total myalgic score, tender point count, depression, and musculoskeletal pain [41]. Additionally, a randomised comparison with duloxetine showed comparable effects on pain and depressive symptoms [42]. Fixed combinations of PEA and ALC have demonstrated improvements in pain and function in rheumatic neuropathies [24] and acute low back pain [25]. Collectively, these controlled studies describe effects on nociceptive and functional outcomes over periods of up to 12 weeks; evidence on administration beyond that period, including the registry analysis [27] and the present cohort, remains observational.

### 4.3. The Dissociation Between Pain and Sleep

The most notable finding of this analysis is not the extent of pain relief but rather the domain in which relief was absent, namely sleep. At six months, 78.1% of completers achieved the minimal clinically important difference for disease impact [34] and 53.1% an increase of at least 5 points on the SF-12 physical component, whereas only 20.3% attained a clinically meaningful improvement in sleep (Table 6, Figure 6); the gradient is the same when all 150 patients are considered with non-responder imputation (37.3%, 23.3% and 10.0%). The PSQI score decreased by only one point on a 0–21 scale (Table 5), leaving nearly all patients above the threshold for poor sleep quality [31]. These instruments were administered only for up to six months, so the dissociation is documented for the first half of the treatment period, and its later course is unknown. Insomnia was also the most frequently recorded adverse event (nine patients): in five of them with a PSQI at six months, the score rose by 0 to 3 points, whereas the median change in the remaining patients was −1 point. Excluding them changes the proportion of PSQI responders only marginally (13/60, 21.7%). Whether the formulation itself worsened sleep in a subset of patients cannot be established from these data, but it is a hypothesis that a prospective study should address with a sleep-specific adverse-event question. This observation is not isolated and aligns with existing knowledge about sleep in this condition. A network meta-analysis of 168 studies found no clear evidence of benefit on sleep for any intervention other than certain forms of exercise, with the certainty of evidence predominantly low to very low [43]. Additionally, the Cochrane review of SNRIs found no difference from placebo in reducing sleep problems, despite effects on pain [14]. Specifically, within the nutraceutical literature, the ALC series from our centre reported no statistically significant change in PSQI at 3 or 6 months, despite significant improvement in VAS, FIQR, and both SF-12 components [18]. Similarly, the fixed-combination study in low back pain found no significant change in emotional parameters, while physical domains improved [25]. This data marks the third time this pattern has been observed. However, the three groups, although made up of different patients, are from the same centre and researchers, so they are not independent replications. A mechanistic interpretation is plausible but speculative. Both compounds act on pathways that influence peripheral and central pain processing more directly than the neurophysiological regulation of sleep: mast-cell stabilisation and glial modulation for PEA, whose relevance to FMS is supported by direct in vivo evidence of cortical glial activation [19,44] and by the broader immune and neuroimmune abnormalities recently reviewed by our group [45], and mitochondrial energy metabolism, neuronal trophism, and mGlu2 up-regulation for ALC [21,46]. A peripheral target also exists, since small-fibre pathology with reduced intraepidermal nerve fibre density is documented in FMS [47], although recent quantitative work indicates that it is considerably milder than in small-fibre neuropathy [48]. Since sleep impairment itself drives pain [49], the persistence of poor sleep may also explain why pain, although substantially reduced, did not return to normal values in most patients. If confirmed prospectively, this has practical implications: patients whose primary complaint is unrefreshing sleep are unlikely to benefit most, and sleep should be addressed by other means rather than expected to improve as a by-product of pain relief. A key methodological warning is necessary. Since every time point on each scale showed *p* < 0.001 (Table 5), statistical significance alone might imply that the domains acted similarly. With 64 paired observations and a consistent change direction, even a one-point shift on a 21-point scale is statistically significant. The difference becomes clear only when the responder analysis against pre-set clinical thresholds [33,34] is considered along with the *p*-values (Table 6). This emphasises a general point about how nutraceutical studies with repeated measures should be reported.

### 4.4. Persistence of the Effect After Treatment Discontinuation

A distinctive feature of this study is that treatment was scheduled for 12 months, whereas the final assessment was performed at 15 months, three months after discontinuation. In the 64 patients who completed the course, pain and disease impact at 15 months were close to the values at 12 months (estimated mean VAS 52.8 versus 52.3, difference +0.5 points, 95% CI −2.4 to +3.5; FIQR 56.8 versus 56.1, +0.7, −1.9 to +3.3; Table 3). The paired analysis detected a small increase in FIQR (+0.7 points, 95% CI +0.3 to +1.2, *p* = 0.004) and a borderline increase in VAS (+0.5, *p* = 0.059), both far below any clinically meaningful threshold (Table 4). This observation applies only to completers, a group selected for having tolerated and continued the treatment for 12 months. Patients who discontinued earlier were not reassessed, so persistence cannot be evaluated in them. Additionally, the interval since their discontinuation (2 to 14 months at the time of the 15-month visit) cannot be used as a wash-out observation. In an uncontrolled study this persistence cannot be separated from the natural course of the disease or from the stability of background therapy. It is nonetheless consistent with the pharmacology of ALC, whose analgesic and antidepressant effects require repeated administration and depend on a transcriptional rather than an acute mechanism. ALC acts as a donor of acetyl groups: it increases the acetylation of the NF-κB p65/RelA subunit and of histone H3 (lysine 27) at the promoter of the Grm2 gene, thereby enhancing the expression of mGlu2 receptors in dorsal root ganglia, spinal cord and forebrain [21,35,36]. In experimental models this epigenetic induction produced effects that outlasted the treatment, being still detectable two weeks after drug withdrawal [36]. Whether a comparable carry-over occurs in patients, and for how long, cannot be established from a single post-treatment visit; a washout phase with repeated assessments would be required, and this is a further element that a prospective study should incorporate.

### 4.5. Safety, Retention and Background Therapy

Adverse events were noted in 10.0% of patients, all of which were mild, and no serious events were recorded during the 12-month treatment period. Additionally, 3.3% of the group stopped treatment due to a recorded adverse event. These numbers only include events recorded in the clinical records during scheduled visits. There was no structured questionnaire for adverse events, patients who stopped treatment were not evaluated afterward, and those lost to follow-up provided no safety data after their last visit. Therefore, the actual incidence might be higher, and these figures should be considered a minimum estimate. The incidence of adverse events is closely comparable to the 13.7% reported for ultramicronised PEA [17] and the 16.7% reported for ALC, where only six out of 183 patients discontinued [18]. These findings align with the absence of major side effects noted in the randomised PEA literature [38,39] and the safety review of PEA, which nonetheless cautioned about the scarcity of data beyond 60 days of treatment [50]. This figure is consistent with those of the two earlier series from the same centre, now over 12 months of treatment; however, uncontrolled comparisons between cohorts cannot establish whether the combination alters the toxicity of either component. Insomnia was the most common adverse event (Section 4.3).

Retention deserves separate comment because it is rarely reported. At the end of the 12-month treatment period, 60.6% (95% CI 52.2–70.3) of patients were estimated to be still on treatment when patients lost to follow-up are censored at their last visit, and 42.7% (35.4–51.4) under the pessimistic assumption that all of them had stopped (Figure 7), the true value lying between these bounds; treatment was not continued beyond 12 months, and the visit at 15 months served only to assess the persistence of the effect after discontinuation. Eighty percent of the discontinuations were attributed to lack of perceived efficacy rather than to adverse events (Table 1), and discontinuations were concentrated in the first three months. For comparison, in the ultramicronised PEA series 57.63% of patients left the treatment for inefficacy [17], and in the pregabalin trials withdrawals for adverse events were about 10% more frequent than with placebo [15]. Retention is a pragmatic composite of efficacy, tolerability and acceptability, and for a supplement taken continuously in a chronic condition it may be a more informative endpoint than a mean change score; it is also the endpoint most directly relevant to the clinical question of whether a nutraceutical is worth starting.

The background treatment for this group was intense: three-quarters of patients were on a tricyclic antidepressant, nearly two-thirds were on a gabapentinoid, and two-fifths were on an opioid at the start. Their treatment plans had to be stable for at least four weeks. Since any increase in background therapy was seen as treatment failure and counted as a discontinuation, patients whose pain got worse could leave the study in two ways: by stopping the formulation or by increasing other drugs. This makes the retention estimate cautious, but it also means that the outcome data after the first month depend on a stable background regimen. Changes in other therapies acted as a time-varying confounder that could not be modeled because they were only noted as the reason for discontinuation. Reductions in rescue medication or background therapy, which would have been useful, were not recorded either. The changes observed happened in patients already taking several centrally acting drugs. Whether this shows an extra pharmacological effect or the natural variability of a condition with changing symptoms cannot be determined without a comparison group. The only controlled evidence for an additional effect is the 12-week randomized study [26].

### 4.6. Strengths and Limitations

The principal strength of this study is its real-life nature combined with duration and breadth: 150 consecutive patients from a tertiary pain centre, selected only by having been prescribed the formulation, with a heavy background therapy and a high symptom burden, followed for 15 months with five validated instruments, safety data, retention data and patient-reported satisfaction. Patients of this kind, on multiple centrally acting drugs, are precisely those most often excluded from randomised trials, and real-world data of this type are increasingly recognised as a necessary complement to trial evidence for questions of long-term effectiveness, tolerability and persistence [51]. A second strength is the transparency of the analysis: three analysis populations are reported side by side (Figure 8 and Figure 9), the primary analysis includes only measurements actually recorded at each visit, the mixed-effects model uses every observed measurement without imputation, and the assumptions about missing data are tested explicitly (Table 7). Thirdly, the breadth of the long-term description: apart from a 24-month registry analysis of 77 patients, which focused on disease-severity questionnaires and included only those who attended all visits [27], no study has tracked patients treated with this combination for more than three months. Additionally, none have reported on pain intensity, sleep, retention, and the course after stopping treatment together. The same real-life design is also the main limitation of the study, as it constrains the inferences that can be drawn. First and most importantly, the study is uncontrolled. In the absence of a comparison group, factors such as the natural history of FMS, regression to the mean, the placebo response, which is substantial in FMS trials [52], concomitant background therapy, and the additional attention associated with follow-up visits all remain plausible contributors to the observed changes, making causal attribution to the formulation impossible.

Second, the product used is a fixed multicomponent formulation that also contains Boswellia serrata, vitamin B6 and vitamin E. Although the doses of these components are low, Boswellia has independent anti-inflammatory activity [53], so the effect observed cannot be ascribed to PEA and ALC alone; for this reason the product is referred to throughout as a multicomponent formulation containing PEA and ALC.

Third, 57.3% of patients did not complete follow-up: 30.0% stopped treatment, mainly due to perceived lack of efficacy, and 27.3% were lost without a documented treatment status (Figure 1). Completers and non-completers did not differ in any baseline clinical characteristic (Table 2), but attrition was partly response-dependent, making the missing-at-random assumption of the mixed model invalid for the entire group. Therefore, we report, along with the mixed model and the LOCF analysis, several missing-not-at-random scenarios (Table 7 and Table S2). LOCF is not conservative here because it uses values recorded while patients were still on treatment [54], and the Cochrane review of pregabalin notes it may overestimate treatment effects [15]. The 12-month change in VAS remained significant even if every non-completer was assumed to have returned to baseline (−13.0 points). However, for FIQR, the change lost statistical significance when discontinuations for inefficacy were assumed to be followed by a worsening of 30 points beyond the last observation. The estimated change should be viewed as a range, from about −13 to −30 points for VAS and from about −5 to −22 points for FIQR at 12 months, with the lower end reflecting pessimistic but plausible assumptions (Figure 8 and Figure 9).

Fourth, changes in additional therapy during follow-up were only noted as treatment failure. Adherence was checked only through patient reports. The Registry does not record disease duration, other painful conditions, use of exercise, physiotherapy, cognitive-behavioural therapy, or opioid doses. Therefore, the response could not be analyzed based on initial severity, disease duration, or existing therapy.

Fifth, sleep and quality of life were measured only up to six months, so the dissociation described here cannot be followed out to 15 months and the insomnia adverse events (Section 4.3) could not be related to a PSQI measured at the same time.

Sixth, the post-treatment observation consisted of a single visit three months after discontinuation, so that the duration of any carry-over effect is unknown. Seventh, the outcome thresholds and the analysis populations were defined by the investigators before this dataset was analysed but were not registered in a public protocol, so their pre-specification cannot be independently verified. Eighth, the within-patient, responder and persistence analyses in the complete-case population describe a self-selected group of patients who tolerated and continued the treatment for 12 months, and therefore give an upper bound of the response. Finally, the study was conducted in a single tertiary centre with extensive experience using these compounds and the results may not be generalisable to other settings.

## 5. Conclusions

In this retrospective, uncontrolled study of 150 fibromyalgia patients, a 12-month add-on treatment with a fixed multicomponent formulation containing palmitoylethanolamide and acetyl-L-carnitine showed reduced pain intensity and disease impact within the first month for those who stayed in the study. Among the 64 patients who completed the treatment, these improvements persisted three months after it ended. Fifty-seven percent of patients did not complete follow-up, and the estimated change at 12 months ranged from about −13 to −30 VAS points, depending on assumptions. Recorded adverse events were mild and infrequent, and between 43% and 61% of patients were still on treatment at the end of the 12-month course. Over six months, sleep quality changed less than other areas, a pattern also seen in previous acetyl-L-carnitine studies from this center.

These results are descriptive. Without a comparison group, they cannot be linked to the formulation, and the high dropout rate limits generalization from the completer analyses. The findings suggest a controlled study should test whether the formulation adds to standard care over at least twelve months, with retention as a co-primary endpoint, continuous sleep measurement, and pre-registered strategies for handling missing data and outcome thresholds.

## Figures and Tables

**Table 3:** Linear mixed-effects models of outcome trajectories, observed-case population.

Timepoint (Months)	Estimated Mean [95% CI]	Difference vs. Baseline [95% CI]	*p*
Pain VAS (0–100)			
Baseline	76.3 [73.7, 78.9]	—	—
1	61.5 [58.8, 64.2]	−14.8 [−17.5, −12.0]	<0.001
2	59.0 [56.2, 61.8]	−17.3 [−20.2, −14.4]	<0.001
3	56.3 [53.4, 59.2]	−20.0 [−23.1, −16.9]	<0.001
6	55.0 [52.0, 58.0]	−21.3 [−24.6, −18.0]	<0.001
9	52.9 [49.8, 56.0]	−23.4 [−26.8, −20.0]	<0.001
12	52.3 [49.1, 55.4]	−24.1 [−27.6, −20.5]	<0.001
15	52.8 [49.6, 56.0]	−23.5 [−27.1, −20.0]	<0.001
FIQR (0–100)			
Baseline	73.6 [71.5, 75.8]	—	—
1	63.0 [60.7, 65.2]	−10.6 [−13.1, −8.2]	<0.001
2	61.0 [58.7, 63.3]	−12.6 [−15.2, −10.1]	<0.001
3	58.3 [55.8, 60.7]	−15.4 [−18.1, −12.6]	<0.001
6	57.3 [54.8, 59.8]	−16.3 [−19.2, −13.5]	<0.001
9	56.5 [53.9, 59.1]	−17.1 [−20.1, −14.1]	<0.001
12	56.1 [53.5, 58.8]	−17.5 [−20.6, −14.4]	<0.001
15	56.8 [54.2, 59.5]	−16.8 [−19.9, −13.7]	<0.001
PSQI (0–21)			
Baseline	10.6 [10.1, 11.0]	—	—
3	9.7 [9.3, 10.2]	−0.8 [−1.2, −0.5]	<0.001
6	9.6 [9.1, 10.1]	−1.0 [−1.3, −0.6]	<0.001
SF-12 PCS			
Baseline	29.2 [27.9, 30.4]	—	—
3	32.8 [31.5, 34.2]	+3.6 [+2.7, +4.6]	<0.001
6	34.0 [32.6, 35.4]	+4.8 [+3.8, +5.8]	<0.001
SF-12 MCS			
Baseline	26.6 [25.3, 27.9]	—	—
3	29.7 [28.3, 31.1]	+3.1 [+2.1, +4.2]	<0.001
6	30.5 [29.0, 31.9]	+3.9 [+2.8, +4.9]	<0.001

Random intercept for patient, timepoint as a categorical fixed effect, and age as a covariate; restricted maximum likelihood (REML) estimation on all available observations. Estimated marginal means with 95% CI (Satterthwaite degrees of freedom); differences versus baseline with 95% CI and *p*-values adjusted for multiplicity by the Dunnett method. Overall effect of time *p* < 0.001 for every outcome (Type III F-test, Satterthwaite degrees of freedom).

## Data Availability

The anonymised dataset supporting the findings of this study is available from the corresponding author on reasonable request.

## Supplementary Materials

The following supporting information can be downloaded at: https://www.mdpi.com/article/10.3390/nu18213693/s1. Figure S1: Individual pain trajectories in the complete-case population (n = 64); Table S1: Number and proportion of missing values per outcome and visit, with the breakdown between patients lost to follow-up and patients who discontinued treatment; Table S2: Sensitivity of the mean change from baseline at 6 and 12 months to assumptions about missing data.

## References

[1] Wolfe F., Clauw D.J., Fitzcharles M.-A., Goldenberg D.L., Häuser W., Katz R.L., Mease P.J., Russell A.S., Russell I.J., Walitt B. (2016). 2016 Revisions to the 2010/2011 fibromyalgia diagnostic criteria. Semin. Arthritis Rheum..

[2] Sarzi-Puttini P., Giorgi V., Atzeni F., Gorla R., Kosek E., Choy E.H., Bazzichi L., Häuser W., Ablin J.N., Aloush V. (2021). Fibromyalgia position paper. Clin. Exp. Rheumatol..

[3] Häuser W., Ablin J., Fitzcharles M.-A., Littlejohn G., Luciano J.V., Usui C., Walitt B. (2015). Fibromyalgia. Nat. Rev. Dis. Primers.

[4] Clauw D.J. (2014). Fibromyalgia: A clinical review. JAMA.

[5] Martini A., Schweiger V., Del Balzo G., Sartori G., Parolini M., Chinellato E., Sarzi-Puttini P., Polati E. (2018). Epidemiological, pharmacological and sociodemographic characterisation of a population of 386 fibromyalgic patients referred to a tertiary pain centre in Italy. Clin. Exp. Rheumatol..

[6] Fitzcharles M.-A., Cohen S.P., Clauw D.J., Littlejohn G., Usui C., Häuser W. (2021). Nociplastic pain: Towards an understanding of prevalent pain conditions. Lancet.

[7] Kosek E., Clauw D., Nijs J., Baron R., Gilron I., Harris R.E., Mico J.-A., Rice A.S.C., Sterling M. (2021). Chronic nociplastic pain affecting the musculoskeletal system: Clinical criteria and grading system. Pain.

[8] Anand P., Dickenson A., Finco G., Marinangeli F., Polati E., Romualdi P., Tzschentke T.M., Canonico P.L. (2019). Novel insights on the management of pain: Highlights from the ‘Science of Relief’ meeting. Pain Manag..

[9] Sarzi-Puttini P., Giorgi V., Marotto D., Atzeni F. (2020). Fibromyalgia: An update on clinical characteristics, aetiopathogenesis and treatment. Nat. Rev. Rheumatol..

[10] Sarzi-Puttini P., Giorgi V., Atzeni F., Gorla R., Kosek E., Choy E.H., Bazzichi L., Häuser W., Ablin J.N., Aloush V. (2021). Diagnostic and therapeutic care pathway for fibromyalgia. Clin. Exp. Rheumatol..

[11] Thieme K., Mathys M., Turk D.C. (2017). Evidenced-based guidelines on the treatment of fibromyalgia patients: Are they consistent and if not, why not? Have effective psychological treatments been overlooked?. J. Pain.

[12] Bidonde J., Busch A.J., Schachter C.L., Overend T.J., Kim S.Y., Góes S.M., Boden C., Foulds H.J.A. (2017). Aerobic exercise training for adults with fibromyalgia. Cochrane Database Syst. Rev..

[13] Moore A., Bidonde J., Fisher E., Häuser W., Bell R.F., Perrot S., Makri S., Straube S. (2025). Effectiveness of pharmacological therapies for fibromyalgia syndrome in adults: An overview of Cochrane Reviews. Rheumatology.

[14] Welsch P., Üçeyler N., Klose P., Walitt B., Häuser W. (2018). Serotonin and noradrenaline reuptake inhibitors (SNRIs) for fibromyalgia. Cochrane Database Syst. Rev..

[15] Derry S., Cording M., Wiffen P.J., Law S., Phillips T., Moore R.A. (2016). Pregabalin for pain in fibromyalgia in adults. Cochrane Database Syst. Rev..

[16] Casini I., Ladisa V., Clemente L., Delussi M., Rostanzo E., Peparini S., Aloisi A.M., de Tommaso M. (2024). A personalized Mediterranean diet improves pain and quality of life in patients with fibromyalgia. Pain Ther..

[17] Schweiger V., Martini A., Bellamoli P., Donadello K., Schievano C., Del Balzo G., Sarzi-Puttini P., Parolini M., Polati E. (2019). Ultramicronized palmitoylethanolamide (um-PEA) as add-on treatment in fibromyalgia syndrome (FMS): Retrospective observational study on 407 patients. CNS Neurol. Disord. Drug Targets.

[18] Schweiger V., Villagrossi L., Taus F., Gottin L., Bonora E., Anderloni M., Varrassi G., Polati L., Nizzero M., Martini A. (2025). Acetyl-L-carnitine as an add-on treatment in fibromyalgia syndrome: A retrospective analysis on 183 patients, according to the generalized linear mixed model for longitudinal data. Biomedicines.

[19] Skaper S.D., Facci L., Giusti P. (2013). Glia and mast cells as targets for palmitoylethanolamide, an anti-inflammatory and neuroprotective lipid mediator. Mol. Neurobiol..

[20] Petrosino S., Di Marzo V. (2017). The pharmacology of palmitoylethanolamide and first data on the therapeutic efficacy of some of its new formulations. Br. J. Pharmacol..

[21] Chiechio S., Caricasole A., Barletta E., Storto M., Catania M.V., Copani A., Vertechy M., Nicolai R., Calvani M., Melchiorri D. (2002). L-Acetylcarnitine induces analgesia by selectively up-regulating mGlu2 metabotropic glutamate receptors. Mol. Pharmacol..

[22] Wang S.M., Han C., Lee S.J., Patkar A.A., Masand P.S., Pae C.U. (2014). A review of current evidence for acetyl-l-carnitine in the treatment of depression. J. Psychiatr. Res..

[23] Veronese N., Stubbs B., Solmi M., Ajnakina O., Carvalho A.F., Maggi S. (2018). Acetyl-L-carnitine supplementation and the treatment of depressive symptoms: A systematic review and meta-analysis. Psychosom. Med..

[24] Parisi S., Ditto M.C., Borrelli R., Fusaro E. (2021). Efficacy of a fixed combination of palmitoylethanolamide and acetyl-l-carnitine (PEA+ALC FC) in the treatment of neuropathies secondary to rheumatic diseases. Minerva Med..

[25] Cominacini M., Valenti M.T., Braggio M., Caramori A., Vedovi E., Dalle Carbonare L. (2025). Unlocking relief: Investigating the impact of a fixed combination of acetyl-L-carnitine and palmitoylethanolamide on traumatic acute low back pain. Eur. J. Neurol..

[26] Salaffi F., Farah S., Sarzi-Puttini P., Di Carlo M. (2023). Palmitoylethanolamide and acetyl-L-carnitine act synergistically with duloxetine and pregabalin in fibromyalgia: Results of a randomised controlled study. Clin. Exp. Rheumatol..

[27] Salaffi F., Lommano M.G., Bianchi B., Farah S., Bandinelli F., Sarzi-Puttini P., Di Carlo M. (2024). Trajectory of Change in the Severity of Symptoms in Patients with Fibromyalgia over 24 Months: Exploratory Analyses of a Combination Pharmacological Intervention. J. Pers. Med..

[28] Vandenbroucke J.P., von Elm E., Altman D.G., Gøtzsche P.C., Mulrow C.D., Pocock S.J., Poole C., Schlesselman J.J., Egger M., STROBE Initiative (2014). Strengthening the Reporting of Observational Studies in Epidemiology (STROBE): Explanation and elaboration. Int. J. Surg..

[29] Bennett R.M., Friend R., Jones K.D., Ward R., Han B.K., Ross R.L. (2009). The Revised Fibromyalgia Impact Questionnaire (FIQR): Validation and psychometric properties. Arthritis Res. Ther..

[30] Salaffi F., Franchignoni F., Giordano A., Ciapetti A., Sarzi-Puttini P., Ottonello M. (2013). Psychometric characteristics of the Italian version of the revised Fibromyalgia Impact Questionnaire using classical test theory and Rasch analysis. Clin. Exp. Rheumatol..

[31] Buysse D.J., Reynolds C.F., Monk T.H., Berman S.R., Kupfer D.J. (1989). The Pittsburgh Sleep Quality Index: A new instrument for psychiatric practice and research. Psychiatry Res..

[32] Ware J., Kosinski M., Keller S.D. (1996). A 12-Item Short-Form Health Survey: Construction of scales and preliminary tests of reliability and validity. Med. Care.

[33] Dworkin R.H., Turk D.C., Wyrwich K.W., Beaton D., Cleeland C.S., Farrar J.T., Haythornthwaite J.A., Jensen M.P., Kerns R.D., Ader D.N. (2008). Interpreting the clinical importance of treatment outcomes in chronic pain clinical trials: IMMPACT recommendations. J. Pain.

[34] Bennett R.M., Bushmakin A.G., Cappelleri J.C., Zlateva G., Sadosky A.B. (2009). Minimal clinically important difference in the fibromyalgia impact questionnaire. J. Rheumatol..

[35] Chiechio S., Copani A., De Petris L., Morales M.E.P., Nicoletti F., Gereau R.W. (2006). Transcriptional regulation of metabotropic glutamate receptor 2/3 expression by the NF-kappaB pathway in primary dorsal root ganglia neurons: A possible mechanism for the analgesic effect of L-acetylcarnitine. Mol. Pain.

[36] Nasca C., Xenos D., Barone Y., Caruso A., Scaccianoce S., Matrisciano F., Battaglia G., Mathé A.A., Pittaluga A., Lionetto L. (2013). L-acetylcarnitine causes rapid antidepressant effects through the epigenetic induction of mGlu2 receptors. Proc. Natl. Acad. Sci. USA.

[37] Schweiger V., Schievano C., Martini A., Polati L., Del Balzo G., Simari S., Milan B., Finco G., Varrassi G., Polati E. (2024). Extended treatment with micron-size oral palmitoylethanolamide (PEA) in chronic pain: A systematic review and meta-analysis. Nutrients.

[38] Lang-Illievich K., Klivinyi C., Lasser C., Brenna C.T.A., Szilagyi I.S., Bornemann-Cimenti H. (2023). Palmitoylethanolamide in the treatment of chronic pain: A systematic review and meta-analysis of double-blind randomized controlled trials. Nutrients.

[39] Viña I., López-Moreno M. (2025). Meta-analysis of palmitoylethanolamide in pain management: Addressing literature gaps and enhancing understanding. Nutr. Rev..

[40] Scuteri D., Guida F., Boccella S., Palazzo E., Maione S., Rodríguez-Landa J.F., Martínez-Mota L., Tonin P., Bagetta G., Corasaniti M.T. (2022). Effects of palmitoylethanolamide (PEA) on nociceptive, musculoskeletal and neuropathic pain: Systematic review and meta-analysis of clinical evidence. Pharmaceutics.

[41] Rossini M., Di Munno O., Valentini G., Bianchi G., Biasi G., Cacace E., Malesci D., La Montagna G., Viapiana O., Adami S. (2007). Double-blind, multicenter trial comparing acetyl l-carnitine with placebo in the treatment of fibromyalgia patients. Clin. Exp. Rheumatol..

[42] Leombruni P., Miniotti M., Colonna F., Sica C., Castelli L., Bruzzone M., Parisi S., Fusaro E., Sarzi-Puttini P., Atzeni F. (2015). A randomised controlled trial comparing duloxetine and acetyl L-carnitine in fibromyalgic patients: Preliminary data. Clin. Exp. Rheumatol..

[43] Imamura M., Robertson C., Hudson J., Whibley D., Aucott L., Gillies K., Beasley M., Stevens M.J., Manson P., Dulake D. (2025). Effects of pharmacological and non-pharmacological interventions for the management of sleep problems in people with fibromyalgia: A multi-methods evidence synthesis. Health Technol. Assess..

[44] Albrecht D.S., Forsberg A., Sandström A., Bergan C., Kadetoff D., Protsenko E., Lampa J., Lee Y.C., Höglund C.O., Catana C. (2019). Brain glial activation in fibromyalgia—A multi-site positron emission tomography investigation. Brain Behav. Immun..

[45] Nizzero M., Schweiger V., Martini A., Gottin L., Varrassi G., Del Balzo G., Secchettin E., Polati L., Coaccioli S., Polati E. (2025). Fibromyalgia syndrome and the immune system: A review with comparative perspectives on chronic immune-related syndromes including CFS/ME and IBS. Explor. Immunol..

[46] Cordero M.D., De Miguel M., Moreno Fernández A.M., Carmona López I.M., Garrido Maraver J., Cotán D., Gómez Izquierdo L., Bonal P., Campa F., Bullon P. (2010). Mitochondrial dysfunction and mitophagy activation in blood mononuclear cells of fibromyalgia patients: Implications in the pathogenesis of the disease. Arthritis Res. Ther..

[47] Üçeyler N., Zeller D., Kahn A.-K., Kewenig S., Kittel-Schneider S., Schmid A., Casanova-Molla J., Reiners K., Sommer C. (2013). Small fibre pathology in patients with fibromyalgia syndrome. Brain.

[48] Falco P., Galosi E., Leone C.M., De Stefano G., Di Pietro G., Di Stefano G., Esposito N., Evangelisti E., Litewczuk D., Mollica C. (2025). Comparative analysis of small nerve fiber density in fibromyalgia syndrome and small fiber neuropathy. Biomedicines.

[49] Finan P.H., Goodin B.R., Smith M.T. (2013). The association of sleep and pain: An update and a path forward. J. Pain.

[50] Gabrielsson L., Mattsson S., Fowler C.J. (2016). Palmitoylethanolamide for the treatment of pain: Pharmacokinetics, safety and efficacy. Br. J. Clin. Pharmacol..

[51] Sherman R.E., Anderson S.A., Dal Pan G.J., Gray G.W., Gross T., Hunter N.L., LaVange L., Marinac-Dabic D., Marks P.W., Robb M.A. (2016). Real-world evidence—What is it and what can it tell us?. N. Engl. J. Med..

[52] Migliorini F., Maffulli N., Eschweiler J., Betsch M., Tingart M., Colarossi G. (2021). Placebo effect in pharmacological management of fibromyalgia: A meta-analysis. Br. Med. Bull..

[53] Bannuru R.R., Osani M.C., Al-Eid F., Wang C. (2018). Efficacy of curcumin and Boswellia for knee osteoarthritis: Systematic review and meta-analysis. Semin. Arthritis Rheum..

[54] Little R.J., D’Agostino R., Cohen M.L., Dickersin K., Emerson S.S., Farrar J.T., Frangakis C., Hogan J.W., Molenberghs G., Murphy S.A. (2012). The prevention and treatment of missing data in clinical trials. N. Engl. J. Med..

