# Supplementary material for: Long-Term Add-On Use of a Fixed Multicomponent Formulation Containing Palmitoylethanolamide and Acetyl-L-Carnitine in Fibromyalgia Syndrome: A 15-Month Retrospective Single-Cohort Study of 150 Patients
Source: Nutrients. 2026 Nov 7;18(21):3693. doi: 10.3390/nu18213693 (PMC13765243; doi:10.3390/nu18213693)
Supplement: Supplementary file 1 [file nutrients-18-03693-s001.zip › nutrients-4598769-supplementary.pdf]

Supplementary Materials

Long-Term Add-On Use of a Fixed Multicomponent Formulation Containing Palmitoylethanolamide and Acetyl-L-Carnitine in Fibromyalgia Syndrome: A 15-Month Retrospective Single-Cohort Study of 150 Patients

Polati E, Anderloni M, Gottin L, Donadello K, Peluso L, Martini A, Polati L, Di Maria V, Villagrossi L, Patton A, Puntillo F, Finco G, Del Balzo G, Schweiger V.

Table S1. Number and proportion of missing values for each outcome at each scheduled visit (N = 150), with the breakdown between patients lost to follow-up and patients who had discontinued treatment.

Visit	Observed, n	Missing, n (%)	Lost to follow-up	Discontinued
Pain VAS (0–100)				
Baseline	150	0 (0.0%)	0	0
Month 1	128	22 (14.7%)	22	0
Month 2	111	39 (26.0%)	35	4
Month 3	92	58 (38.7%)	35	23
Month 6	80	70 (46.7%)	37	33
Month 9	71	79 (52.7%)	40	39
Month 12	64	86 (57.3%)	41	45
Month 15	64	86 (57.3%)	41	45
FIQR (0–100)				
Baseline	150	0 (0.0%)	0	0
Month 1	127	23 (15.3%)	23	0
Month 2	111	39 (26.0%)	35	4
Month 3	91	59 (39.3%)	35	24
Month 6	80	70 (46.7%)	37	33
Month 9	71	79 (52.7%)	40	39
Month 12	64	86 (57.3%)	41	45
Month 15	64	86 (57.3%)	41	45
PSQI (0–21)				
Baseline	150	0 (0.0%)	0	0
Month 3	91	59 (39.3%)	35	24
Month 6	80	70 (46.7%)	37	33
SF-12 PCS				
Baseline	150	0 (0.0%)	0	0
Month 3	91	59 (39.3%)	35	24
Month 6	80	70 (46.7%)	37	33
SF-12 MCS				
Baseline	150	0 (0.0%)	0	0
Month 3	91	59 (39.3%)	35	24
Month 6	81	69 (46.0%)	36	33

VAS, visual analogue scale; FIQR, revised Fibromyalgia Impact Questionnaire; PSQI, Pittsburgh Sleep Quality Index; SF-12 PCS and MCS, Physical and Mental Component Summary of the 12-item Short Form Health Survey. Missing values are counted among patients without an observed assessment at the visit; "lost to follow-up" denotes patients with no documented treatment status whose last visit preceded the one considered (n = 41 overall), and "discontinued" denotes patients with a documented discontinuation before the visit (n = 45 overall). Values carried forward in the source dataset after a documented discontinuation are not counted as observed. No patient who completed treatment (n = 64) has a missing value at any visit. The PSQI and the SF-12 were administered at baseline, 3 and 6 months only. Adverse events were recorded for every patient (an empty field denoting no event), whereas the treatment satisfaction score is missing for 35 patients, all of them non-completers.

Table S2. Sensitivity of the mean change from baseline in VAS and FIQR at 6 and 12 months to assumptions about missing data.

Scenario	n (6 / 12 months)	VAS, 6 months	VAS, 12 months	FIQR, 6 months	FIQR, 12 months
Observed cases at the visit	80 / 64	−25.5 [−29.0, −22.0]	−30.5 [−34.0, −27.1]	−19.5 [−22.7, −16.3]	−22.1 [−25.9, −18.4]
LOCF for discontinued patients	113 / 109	−18.2 [−21.7, −14.7]	−19.4 [−23.1, −15.8]	−13.3 [−16.2, −10.3]	−13.2 [−16.4, −10.1]
Delta-adjusted LOCF, δ = 5	113 / 109	−17.2 [−20.8, −13.5]	−18.0 [−21.9, −14.1]	−12.1 [−15.2, −8.9]	−11.6 [−15.0, −8.1]
Delta-adjusted LOCF, δ = 10	113 / 109	−16.2 [−20.0, −12.3]	−16.6 [−20.8, −12.4]	−10.9 [−14.3, −7.4]	−10.0 [−13.7, −6.2]
Delta-adjusted LOCF, δ = 15	113 / 109	−15.4 [−19.4, −11.3]	−15.4 [−19.8, −11.0]	−9.7 [−13.5, −6.0]	−8.4 [−12.5, −4.4]
Delta-adjusted LOCF, δ = 20	113 / 109	−14.6 [−18.8, −10.4]	−14.3 [−19.0, −9.6]	−8.8 [−12.8, −4.8]	−7.1 [−11.5, −2.7] (p = 0.004)
Delta-adjusted LOCF, δ = 25	113 / 109	−14.1 [−18.4, −9.7]	−13.5 [−18.4, −8.7]	−7.9 [−12.1, −3.7]	−5.8 [−10.5, −1.2] (p = 0.034)
Delta-adjusted LOCF, δ = 30	113 / 109	−13.6 [−18.1, −9.1]	−12.8 [−17.9, −7.8]	−7.2 [−11.6, −2.8] (p = 0.003)	−4.9 [−9.8, 0.1] (p = 0.109)
BOCF for all discontinued patients	113 / 109	−18.1 [−21.3, −14.8]	−17.9 [−21.4, −14.4]	−13.8 [−16.6, −11.0]	−13.0 [−16.0, −10.0]
BOCF for all non-completers (worst case)	150 / 150	−13.6 [−16.4, −10.8]	−13.0 [−15.9, −10.2]	−10.4 [−12.7, −8.1]	−9.4 [−11.8, −7.1]

Observed cases at the visit: all patients with an assessment at that visit (6 months: n = 80; 12 months: the 64 completers). Mean change from baseline with 95% CI (t-based); Wilcoxon signed-rank p < 0.001 unless stated. LOCF, last observation carried forward (the last value recorded before discontinuation); δ, number of points added to the carried-forward value of each patient who discontinued for lack of perceived efficacy (n = 36), the other discontinued patients keeping their carried-forward value; BOCF, baseline observation carried forward. Patients lost to follow-up (n = 41) contribute only the assessments actually performed, except in the worst-case scenario, in which they are assumed to have returned to baseline. For VAS and FIQR the tipping point, i.e. the smallest δ at which the mean change was no longer statistically significant, was not reached for VAS at either visit, nor for FIQR at 6 months, and was reached at δ = 30 for FIQR at 12 months. Imputation applies only to patients without an observed value at the visit considered; patients who discontinued after that visit contribute their observed value.

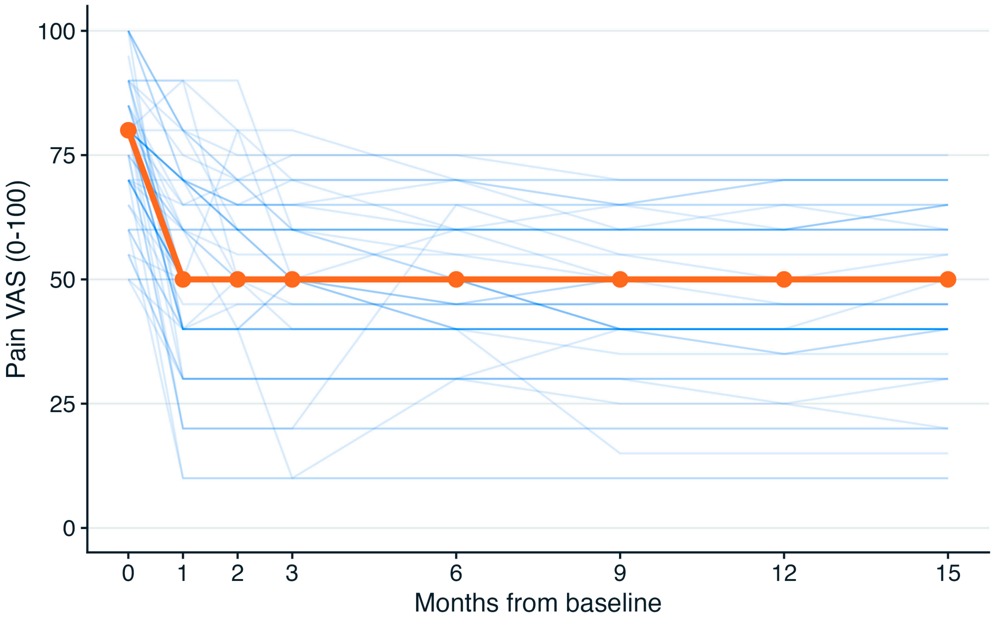

Each thin line is one patient in the complete-case population (n = 64); the orange line is the median of this population. The spread shows how heterogeneous the response is, which the median alone conceals.

Figure S1. Individual pain trajectories in the complete-case population (n = 64). Each thin line is one patient; the orange line is the cohort median. The dispersion shows how heterogeneous the individual response is, which the median alone conceals.
